# Polyelectrolyte Multilayered Capsules as Biomedical Tools

**DOI:** 10.3390/polym14030479

**Published:** 2022-01-25

**Authors:** Ana Mateos-Maroto, Laura Fernández-Peña, Irene Abelenda-Núñez, Francisco Ortega, Ramón G. Rubio, Eduardo Guzmán

**Affiliations:** 1 Departamento de Química Física, Facultad de Ciencias Químicas, Universidad Complutense de Madrid, Ciudad Universitaria s/n, 28040 Madrid, Spain; ana.mateos@ucm.es (A.M.-M.); laura.fernandez.pena@ucm.es (L.F.-P.); irenabel@ucm.es (I.A.-N.); fortega@quim.ucm.es (F.O.); rgrubio@quim.ucm.es (R.G.R.); 2 Max Planck Institute for Polymer Research, Ackermannweg 10, 55128 Mainz, Germany; 3 Centro de Espectroscopía y Correlación, Universidad Complutense de Madrid, Ciudad Universitaria s/n, 28040 Madrid, Spain; 4 Instituto Pluridisciplinar, Universidad Complutense de Madrid, Paseo Juan XXIII 1, 28040 Madrid, Spain

**Keywords:** biomedical, capsules, drug delivery, layer-by-layer, multilayers, polyelectrolyte, controlled release

## Abstract

Polyelectrolyte multilayered capsules (PEMUCs) obtained using the Layer-by-Layer (LbL) method have become powerful tools for different biomedical applications, which include drug delivery, theranosis or biosensing. However, the exploitation of PEMUCs in the biomedical field requires a deep understanding of the most fundamental bases underlying their assembly processes, and the control of their properties to fabricate novel materials with optimized ability for specific targeting and therapeutic capacity. This review presents an updated perspective on the multiple avenues opened for the application of PEMUCs to the biomedical field, aiming to highlight some of the most important advantages offered by the LbL method for the fabrication of platforms for their use in the detection and treatment of different diseases.

## 1. Introduction

From the seminal works dealing with the fabrication of polyelectrolyte multilayered capsules (PEMUCs) [1,2,3,4,5] to date, the optimization of the fabrication procedures and development of their potential applications have stimulated a very important piece of research, which allows including PEMUCs among the most exploited objects of polymer nanotechnology [6]. The extensive development of LbL materials for the fabrication of capsules takes advantage of the simplicity and versatility offered for this methodology, combined with their low cost and modularity. This has made possible the exploitation of LbL multilayered nanostructures for the fabrication of functional materials with controlled thickness and composition, and tunable properties and structure, which can be used in a broad range of technological fields, including food and cosmetic industries, or the fabrication of different biomedical devices [7,8,9,10,11,12,13]. The widespread use of PEMUCs, especially in the biomedical field, has been also stimulated by the absence of shape and size restrictions offered by the LbL method for manufacturing capsules by combining different assembled blocks interacting through different types of interactions. Therefore, the existence of a true electrostatic interaction is not necessary for the fabrication of PEMUCs, allowing the fabrication of capsules with molecules of very different natures, i.e., the LbL method is not limited only to polyelectrolytes [14]. Furthermore, the possibility to perform the assembly process in aqueous media at room temperature is a very important aspect for the application of the LbL method, especially when the biomedical applications of the obtained materials are considered [15,16,17]. This allows minimizing the possible degradation of the bioactive properties of the used molecules [11]. 

PEMUCs contain two well-defined compartments: The cavity and the polyelectrolyte shell. The semipermeable character of the polyelectrolyte shell provides a suitable environment enabling the permeation of encapsulated molecular compounds, with relatively low molecular weight (less than 1 kDa), and ions inside the cavity, avoiding the permeation of compounds with high molecular size [18]. This allows the use of PEMUCs for loading in a protected environment and for delivery of a broad range of substances, e.g., inorganic nanoparticles, carbon nanotubes, antibodies, dyes, quantum dots, antitumoral drugs, proteins, or nucleic acids [19,20,21,22,23,24,25,26], which contributes to the enhanced bioavailability and solubility of active compounds [27,28]. This has stimulated the exploitation of polyelectrolyte multilayered capsules as cargo platforms in several biomedical applications inside the bloodstream [29,30,31]. Furthermore, these micro-/nanocontainers offer a very interesting capacity for controlled stimulus-sensitive release of the encapsulated functional components in response to specific external physical (ultrasound, magnetic field, laser pulse, or optical radiation), chemical (pH, ionic strength, or polarity of the environment), or biochemical (receptors or target cells) stimuli [32,33,34,35,36]. Therefore, it is possible to exploit PEMUCs as targeted drug delivery systems or for designing treatments with prolonged action through a controlled release of the encapsulated drugs [37,38]. Furthermore, PEMUCs can be functionalized with a broad range of components enabling the fabrication of complex theranostics systems [39,40], or sensors for determining the concentration of specific molecules or the pH of the medium [41].

This review presents an updated overview on the different possibilities offered for LbL PEMUCs in the biomedical field, with the aim of highlighting the advantages provided by the LbL method in the assembly of novel materials for biomedical applications.

## 2. Assembly Methodologies for the Fabrication of PEMUCs

This section is intended to provide a general overview of the state of the art of the fabrication of PEMUCs by exploiting the LbL method, and presents a general description of the most common methodological approach for obtaining hierarchical LbL capsules. A more complete perspective on the manufacturing of LbL material can be found in previous publications [14,42,43].

### 2.1. Immersive Assembly: Dip-Coating Deposition on Flat Surfaces

**Figure 1:**
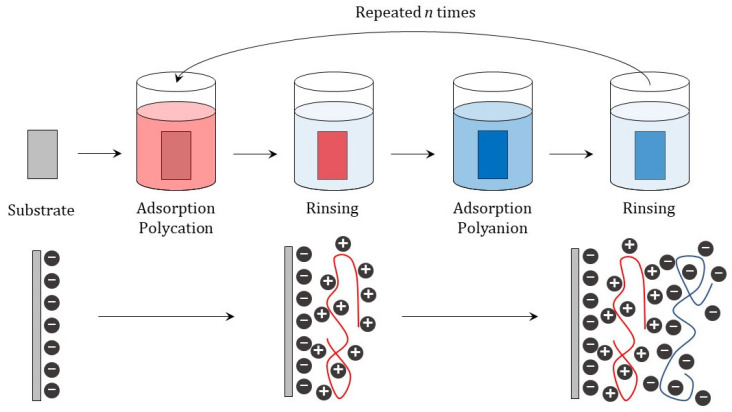
Sketch representing the use of dip-coating deposition for the fabrication of LbL materials using a negatively charged flat substrate as template. Reprinted from Mateos-Maroto et al. [42], with permission under Open access CC BY 4.0 license, https://creativecommons.org/licenses/by/4.0/ (accessed 20 January 2021).

The most common method used for LbL assembly continues to be the dip-coating deposition, or some of their adaptions [7,14,43,44,45,46,47,48]. This method offers a very simple and versatile alternative for coating flat or non-flat substrates, or even substrates with very complex geometries. The approach presents two main drawbacks: The long time required for material fabrication, and the high quantities of material demanded for the fabrication of each coating layer. In brief, the deposition of a multilayer by alternate dip-coating relies on the alternate exposure of the substrate to solutions containing the mutual interacting molecules that will form the multilayer, i.e., the layering solutions, with intermediate washing cycles between the deposition of adjacent layers to avoid cross-contamination phenomena [42,47,49,50]. This method has been traditionally used for the assembly of LbL materials using flat macroscopic substrates as templates, providing an important guide for optimizing the assembly of LbL films on more complex geometries. This is very important for the optimization of capsule fabrication processes, which commonly require the use of colloidal objects as a template. Figure 1 presents a sketch of the dip-coating methodology for the fabrication of LbL materials.

### 2.2. Towards the Fabrication of Hollow Capsules: Immersive Assembly on Colloidal Templates

The adaption of the immersive deposition approach to the fabrication of PEMUCs using colloidal templates that are generally dissolved or dispersed in a liquid, normally water, is not trivial, and requires additional steps to ensure an effective separation of the assembled systems from the excess of non-adsorbed material. This is possible by pelleting the decorated colloids by combining, during the washing process, the separation of the unbound and assembled material, commonly using centrifugation, with the redispersion in the solvent [1,2,3,4,51,52,53,54]. Thus, after the deposition of each layer, the polymer-decorated colloids are settled at the bottom of the tube by centrifugation, and the supernatant containing the excess of unbound material is removed. Afterwards, the decorated colloids are redispersed in the solvent, and the cycle including the sedimentation and redispersion is repeated several times (normally three times) to ensure the complete removal of the excess of unbound material from the dispersion. This allows the deposition of a new layer by repeating the adsorption + washing sequence [55,56]. However, the scalability of the above-described immersive process is commonly limited by the long time required for the fabrication process and its labor-intensive character [57].

**Figure 2:**
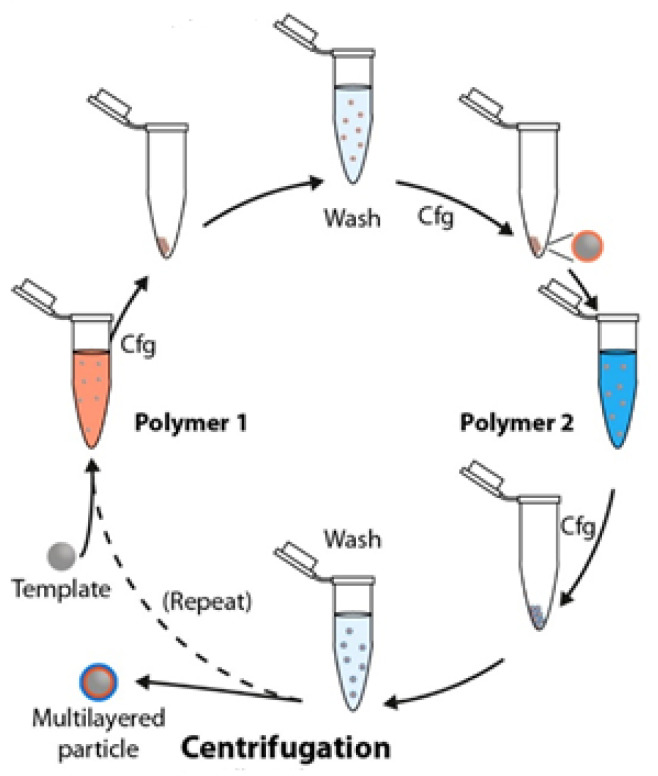
Schematic representation of the most common methodologies used for fabricating PEMUCs using colloidal particles as templates. Reprinted from Yan et al. [52], Copyright (2014), with permission from American Chemical Society.

The above-described approach for the fabrication of PEMUCs using colloidal templates requires an additional step in the template (sacrificial template) dissolution for the fabrication of the final capsule (hollow capsules). The dissolution process is commonly achieved by a chemical treatment that depends on the specific chemistry of the colloidal template. Thus, fluoride acid is used for dissolving silicon dioxide templates, diluted hydrochloride acid when melamine formaldehyde resins are used, and tetrahydrofuran for polystyrene latex templates [58]. In recent years, the use of calcium carbonate particles as templates has gained relevance. This requires the use of ethylene-diamine-tetracetic acid for ensuring the template dissolution [29,31]. It is worth mentioning that the preparation of PEMUCs using sacrificial colloidal particles as templates is not recommended for specific application because the obtained capsules can retain some toxic residues from the templates (dirty capsules), or solvent traces. Figure 2 presents a scheme representing the different steps involved in the fabrication of PEMUCs using colloidal particles as templates.

The dip-coating methodology, and its adaption for fabricating LbL films on the surface of colloidal templates, offer a very simple approach for fabricating PEMUCs. However, as was stated above, it requires the use of centrifugation for separating the polymer-decorated colloidal particles and the excess of unbound material, which can force the aggregation of the obtained capsules, especially when high rotation speeds are used, making it very difficult to apply this methodology for templates with nanometric size, or lower density than the water. Furthermore, the inclusion of centrifugation steps reduces the possibilities for automatization and scaling of the fabrication procedure, which can be partially solved using the serum replacement method for the separation step, thus helping reduce the aggregation of the obtained capsules [59].

The necessity of avoiding the centrifugation steps, or more specifically the separation methodology, is a very important driving force of the development of new approaches for immersive LbL deposition on colloidal substrates. It should be noted that the necessity to introduce separation steps emerges from the use of layering solutions with concentrations exceeding those necessary for the saturation of the substrate surface [53,60]. However, the separation methodologies can be removed from the assembly protocol by controlling carefully the number of layering species, and by adding only the amount required for the saturation of the surface of the colloidal template, which provides an increase in the speed of the assembly process by a factor of 3 [3,61]. This requires a careful evaluation of the dependence of the surface charge, normally evaluated in terms of the zeta potential, and the concentration of the layering solution, to avoid the aggregation of the obtained capsules. The latter can be also partially prevented by sonication of the dispersion during the assembly process [61,62,63,64].

The use of colloidal objects lighter than water as templates, e.g., emulsion droplets, vesicles, or liposomes, introduces additional problems to the separation of the obtained capsules and the excess of unbound materials, making it necessary to significantly modify the assembly protocol. These modifications include the use of creaming/skimming cycles instead of centrifugation/redispersion to ensure the separation of the coated droplets from the excess unbound material when emulsion droplets are used as templates. Thus, the lower density of the emulsion droplets in relation to the aqueous continuous phase facilitates their recovery by flotation in the layering solutions [65,66], and the use of centrifugation can be exploited to obtain an enhanced creaming yield [67,68,69]. Conversely, the separation of the excess unbound material when vesicles or liposomes are used as templates for the LbL assembly becomes even more complex, involving in some cases up to three different steps for each pair of deposited bilayers [70,71]: (i) The first layering solution is added to a diluted suspension containing vesicles/liposomes to form the first layer; (ii) the second layering solution is added to the dispersion containing the polyelectrolyte-decorated vesicles/liposomes, and the excess unbound material resulting after the deposition of the first layer, leading to the formation of the second layer and interpolyelectrolyte complexes; and (iii) the interpolyelectrolyte complexes are settled by centrifugation, and a dispersion containing the polyelectrolyte-decorated vesicles/liposomes is obtained. It should be noted that after the deposition of the first bilayer, the deposition of the subsequent bilayers can be obtained following a similar sequence. However, this procedure limits the maximum number of bilayers that can be deposited to five or six because, during the separation step, the formed interpolyelectrolyte complexes can interact with the obtained capsules, leading to a loss in capsules during the centrifugation. The loss in capsules has been estimated to be around 5% of the total per deposited bilayer [70].

### 2.3. Deposition Assisted by Magnetic Fields

Magnetic fields can be used for controlling the layering process, or moving magnetic colloids in and out of the layer solution [40]. Therefore, the use of magnets is another very interesting methodology for ensuring the separation of the excess unbound molecules from the assembled capsules [40], making it necessary to load the capsules, commonly with lower density than water, with magnetic nanoparticles. This approach has been exploited for the separation of emulsion droplets coated by LbL films [72] or LbL films deposited on magnetic colloids [73], allowing a recovery close to 100% of the obtained capsules.

It should be noted that the use of deposition assisted by magnetic fields is far from being a general methodology. However, the possibility of handling the substrate by the application of a magnetic field provides an interesting perspective for the assembly of PEMUCs, and especially when small colloids are used as templates.

### 2.4. Deposition on Immobilized Colloids

**Figure 3:**
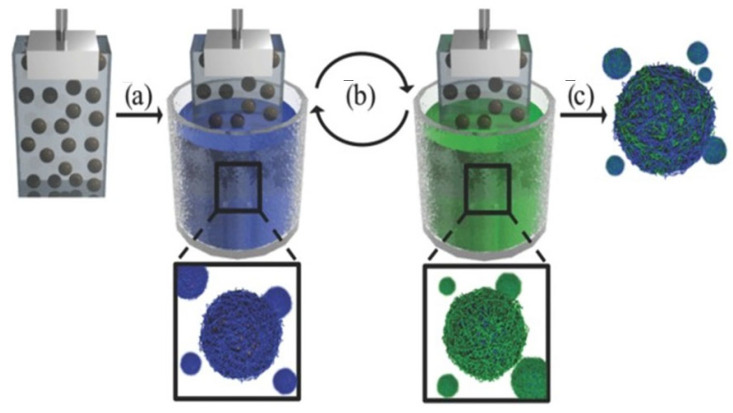
Sketch of the fabrication of PEMUCs using immobilized colloids as templates. The different letters indicate the different steps of the process. (**a**) Immersion of the immobilized colloid into the first layering solution and subsequent rinsing (the rinsing step is not shown for simplicity). (**b**) Immersion on the second layering solution and subsequent rinsing. (**c**) The coated colloids are recovered from the agarose matrix. The steps (**a**,**b**) are repeated until the desired number of layers is obtained. Reprinted from Richardson et al. [74], Copyright (2013), with permission from John Wiley and Sons.

The complicated separation steps can be avoided by fixing the colloidal templates in immobilization matrices, e.g., agarose hydrogels, in such a way that allows considering the systems as a planar substrate. This reduces the layer deposition on the colloids to a simple dip-coating procedure, which is followed, after the deposition of the desired number of layers, by the erosion of the immobilization matrix by heating at 37 °C. The final step for obtaining a clean dispersion containing the PEMUCs relies on the separation of the agarose and the capsules by at least three cycles of centrifugation+redispersion in the aqueous medium. This methodology allows the automatization of the assembly process of LbL films on colloidal templates [74]. Figure 3 represents a sketch showing a typical process of fabrication of LbL PEMUCs using immobilized templates. 

It is worth mentioning that the design of automatized methodologies for LbL assembly is one of the current challenges towards the industrial scaling of the fabrication of PEMUCs, for which several promising attempts have been made [43,74,75,76]. An example of automatization emerged from the work by Peiffre et al. [77]. They introduced a computer-controlled device allowing for the fabrication of up to 1000 polyelectrolyte layers on colloidal particles with a diameter of about 100 μm. Another very interesting alternative towards the automatization is the use of a porous matrix that is filled with polyelectrolyte molecules and colloidal particles by the application of pressure. This allows fabricating a broad range of supramolecular LbL structures, including nanotubes, after the dissolution of the matrix [78].

### 2.5. Towards the Scalability of the Fabrication of PEMUCs

**Figure 4:**
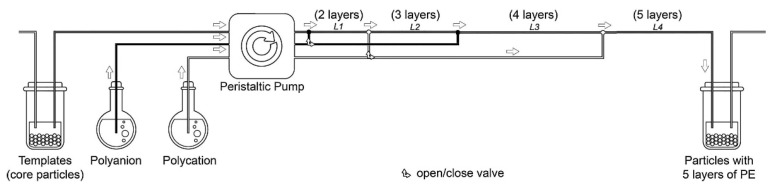
Sketch representing the experimental approach for the assembly process of LbL PEMUCS using a tubular reactor. Reprinted from Elizarova et al. [80], Copyright (2016), with permission from Elsevier.

The solvent-induced precipitation emerges as a very promising alternative for facilitating the separation of the excess unbound material [79], avoiding the use of centrifugation. This approach increases the assembly velocity and the recovery yield, which are important issues for the scaling up of the fabrication process of PEMUCs. The scalability of the fabrication of PEMUCs can be achieved by performing the assembly in tubular flow reactors, which allows the fabrication of capsules with a fixed number of layers through a continuous process. However, the use of this type of process has an important drawback associated with the retention of small amounts of the last deposited polyelectrolyte in the medium, which can lead to cross-contamination during the preparation of the capsules [80]. Figure 4 presents a sketch of the experimental flow for the fabrication of PEMUCs using a tubular reactor.

### 2.6. New Avenues on the Fabrication of PEMUCs: Microfluidic Approaches

**Figure 5:**
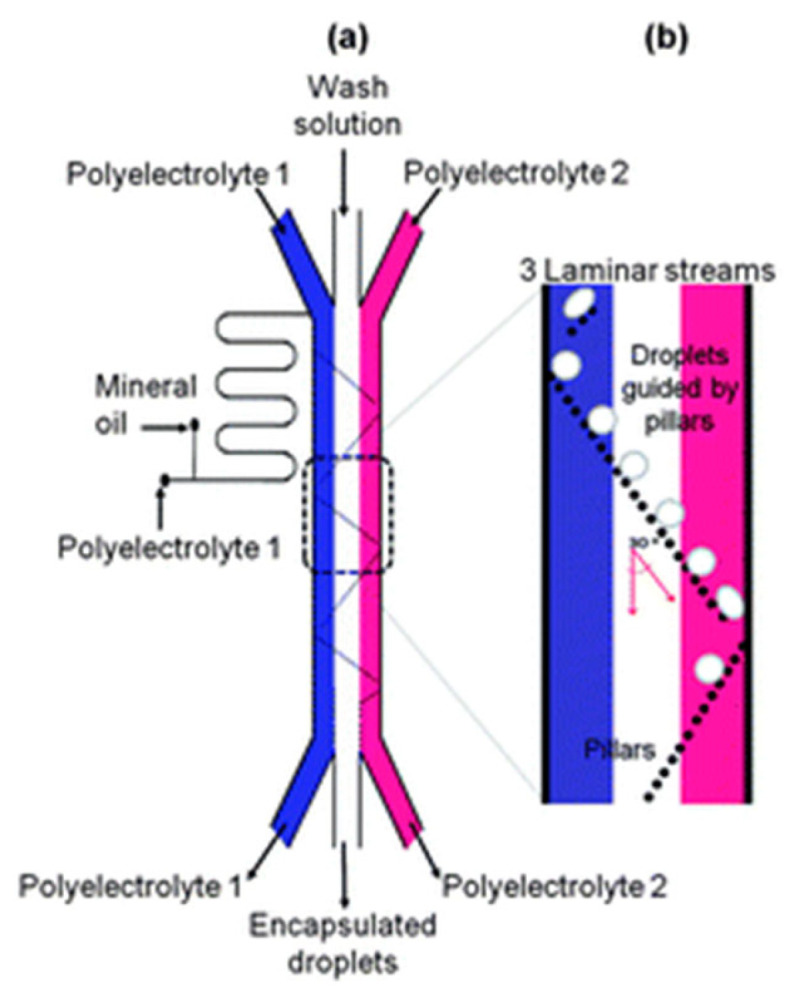
Sketch representing an experimental configuration for the assembly of LbL PEMUCS using a microfluidic device. (**a**) General view representing the inputs and outputs of the assembly process. (**b**) Expanded view of the process of deposition of one bilayer. Reprinted from Kantak et al. [88], Copyright (2011), with permission from The Royal Society of Chemistry.

The use of microfluidic approaches for fabricating PEMUCs has gained importance in recent years [76,81,82,83], contributing to the reduction in the number of aggregation processes during the assembly process [84,85,86,87,88]. Microfluidic techniques offer multiple possibilities for fabricating LbL capsules, with the alternate displacement of the layering and washing solutions within the microfluidic chip by the application of pressure or vacuum being the most extended [89,90,91]. Thus, it is possible to coat the particulate template by exposing the particle flow stream to the layering or washing solutions flowing in a perpendicular direction [85]. The alternate exposure of the colloidal template to the polyelectrolyte and washing streams is another possibility for the fabrication of PEMUCs using microfluidic methods [88]. Figure 5 shows a sketch of one of the possible configurations for fabricating LbL PEMUCs using a methodology using a microfluidic device.

A fluidized bed can be also exploited for the fabrication of PEMUCs using colloidal particles as templates [92]. This type of methodology exploits the upward force of the washing or layering solutions that counteract the gravitational forces driving particle sedimentation. This leads to a situation in which particles are lifted to form a fluidized bed, which allows the fabrication of PEMUCs on substrates having a diameter of up to 3 μm [93]. It should be noted that although the use of microfluidic approaches for the fabrication of PEMUCs provides very interesting perspectives, their current implementation remains rather limited because of the high cost associated with the required instrumentation and the implementation of each individual process [57].

## 3. A Brief Introduction to the Physico-Chemical Aspects Driving the Formation of LbL Polyelectrolyte Multilayers

The optimization of the fabrication processes of PEMUCs using the LbL methods requires the understanding of fundamental aspects governing the assembly of multilayered films. This is important because modulating the fabrication processes of PEMUCs to obtain systems with ad hoc designed properties and functionalities is only possible with a deep understanding of the physico-chemical-based underlying the assembly process of LbL materials [14,94]. This section focuses on providing a brief description of the foundations of the LbL assembly.

### 3.1. Growth of Polyelectrolyte Multilayers

The ability of polyelectrolytes to assemble in self-organized supramolecular structures, in addition to their capacity to form interpolyelectrolyte complexes upon their mixing with oppositely charged polyelectrolytes, play a central role in the control of the fabrication processes of LbL materials [44,46,95,96,97], allowing the fabrication of LbL multilayered films in which the thickness can be tuned almost at will. This is important for the optimization of different properties associated with the potential of LbL materials, e.g., transparency, retention of encapsulated drugs or wetting properties, and adhesion. 

**Figure 6:**
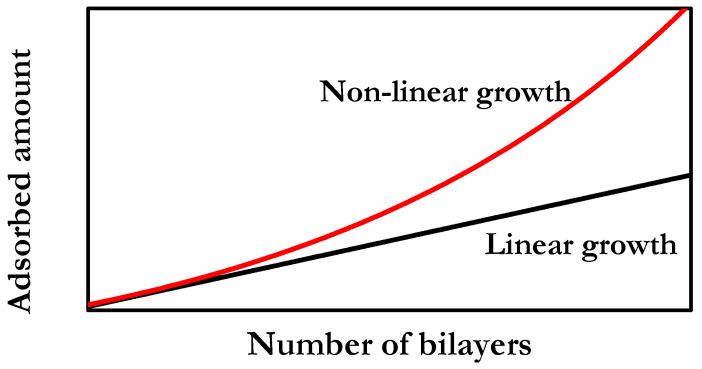
Idealized representation of the dependences of the adsorbed amount on the number of bilayers for LbL polyelectrolyte multilayers undergoing linear and non-linear growth. Reprinted from Mateos-Maroto et al. [42], with permission under Open access CC BY 4.0 license, https://creativecommons.org/licenses/by/4.0/ (accessed 20 January 2022).

It is now well accepted that the adsorbed amount (or thickness) of LbL films can have two different dependences on the number of deposited bilayers. This is strongly dependent on the specific pair of molecules assembled in the multilayer and the conditions used for the multilayer assembly, which can drive the emergence of two different types of multilayer growths: Linear and non-linear. The latter is commonly defined as exponential growth, even though the dependence of the adsorbed amount on the number of deposited bilayers is not always strictly exponential. In the following, the multilayers are defined following the notation (A − B)_n_, with A and B representing the species forming each layer, and the subindex n accounting for the number of deposited bilayers. Figure 6 shows a general representation of the most common growth emerging in LbL polyelectrolyte films.

Linear growth is characterized by a quasi-linear increase in the adsorbed amount on the number of deposited bilayers. This means that the adsorbed amount is constant for each bilayer, which leads to the growth in the multilayer of only a few nanometers after the deposition of a single bilayer. This thickness increase is approximately the sum of the characteristic lengths of the deposited polyelectrolytes, i.e., polycation and polyanion. Some examples of multilayers with linear growth are (PAH-PSS)_n_ (where PAH and PSS are poly(allylamine hydrochloride) and poly(4-styrenesulfonate of sodium), respectively), and (PDADMAC-PSS)_n_ (where PDADMAC is poly(diallyldimethylammonium chloride) under conditions in which the polyelectrolytes present a high charge density [98,99]. There are other examples of multilayers presenting linear growth, e.g., (PAH-PAA)_n_ and (PM2VP-PSS)_n_ (where PAA and PM2VP are poly(acrylic acid) and poly(*N*-methyl-2-vinyl pyridinium chloride), respectively) [100]. By comparison, non-linear growth is characterized by an increase in the deposited amount per bilayer that is faster than in the case of linear growth, which leads to a supralinear dependence of the adsorbed amount on the number of deposited bilayers. This means that the bilayer thickness does not remain constant per bilayer, and hence it is not directly related to the characteristic length of the layering polymers. Non-linear growth is frequently found in LbL films containing biopolymers (normally polypeptides or polysaccharides) e.g., (CHI-PAA)_n_, (PLL-HA)_n_ or (PLL-PGA)_n_ (with CHI, PLL, HA and PGA being chitosan, poly(l-lysine), hyaluronic acid and poly(glutamic acid), respectively) [101,102,103,104,105]. Furthermore, non-linear growth can also be found in multilayers containing synthetic polymers when the effective charges of the polyelectrolytes are relatively low, e.g., (PDADMAC-PSS)_n_ multilayers assembled from solutions with high ionic strengths or using solvents of reduced polarity [106,107,108,109]. This evidences that the thickness and growth dependence of polyelectrolyte multilayers can be tuned by changing the assembly conditions. In addition to the above discussed dependences of the adsorbed amount on the number of deposited bilayers, it should be mentioned that for specific combinations of polyelectrolyte pairs, exotic growth dependences can emerge. In this case, the multilayer growth occurs without respecting the general rules guiding the LbL assembly [110,111].

The fabrication of LbL films is a well-established research field with more than three decades of effort trying to elucidate the main rules governing the assembly process. However, to date, the origin of the different types of dependences of the adsorbed amount on the number of bilayers is far from clear. A critical discussion about the different perspectives that attempts to explain the different types of growth in LbL films can be found in our previous works [14,42].

### 3.2. Charge Balance in Polyelectrolyte Multilayers: Inversion and Compensation

The electrostatically driven self-assembly of LbL multilayers emerges from a direct electrostatic interaction between oppositely charged polyelectrolytes in adjacent layers. However, this picture is an oversimplification, and a true description of the interaction balance requires the inclusion of a broad range of interactions between the different components of the systems, which in many cases do not present a strictly electrostatic origin [108,112]. Therefore, the description of the assembly of LbL materials must consider the intricate balance between different types of interactions, which depends on two different aspects: (i) Solvent quality for the polyelectrolytes (ionic strength, pH, or temperature), and (ii) competition between electrostatic and entropic contributions [94,106,113].

It is commonly accepted that the assembly of polyelectrolytes, and many other charged entities, onto oppositely charged surfaces is driven by charge inversion phenomena, i.e., the adsorption is not stopped after the neutralization of the charge of the surface, and progressed until the initial surface adopts a charge with the same sign as that of the layering molecule [114]. This results from the contribution of the steric hindrance, which makes it very difficult to obtain a perfect mismatch between the charges of the adsorbing species and those existing on the surface. This leads to the adsorption of additional molecules in relation to the number that is required to ensure the neutralization of the surface, and hence the formation of a polyelectrolyte layer on an oppositely charged surface is associated with charge inversion phenomena. This results in the formation of fuzzy layers containing multiple polyelectrolyte segments (loops, tails, and trains) protruding into the liquid environment surrounding the film. Therefore, an overcompensation of the charge of the bare surface after the deposition of the polyelectrolyte may be expected, which is self-limited due to the repulsions between the adsorbed molecules and those with the same sign remaining in solution. The addition of additional layers to the LbL film occurs following a similar mechanism, in which the overcompensation plays a central role in governing the multilayer growth [104,106,115,116]. 

**Figure 7:**
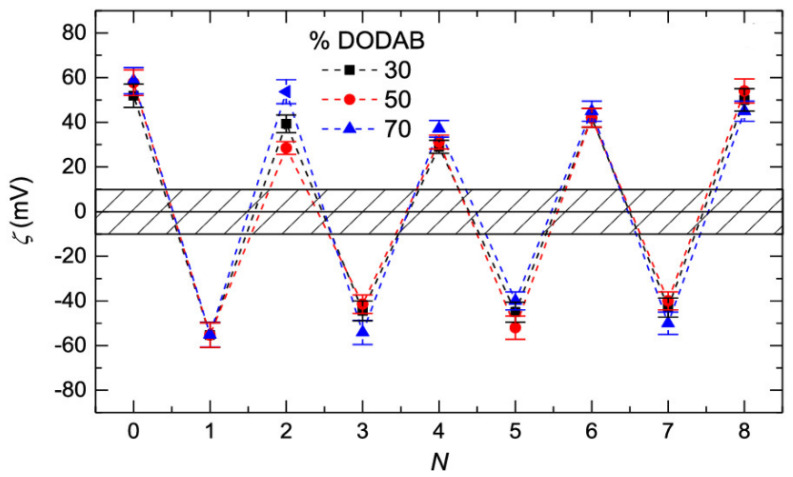
Change in the ξ potential with the alternate deposition of PAH and PSS layers onto positively charged liposomes with different charge density (indicated by %DODAB) from polyelectrolyte solutions with concentration 1 g/L, and ionic strength fixed at 10 mM. Reprinted from Mateos-Maroto et al. [71], Copyright (2021), with permission from American Chemical Society.

The most common approach for evaluating the emergence of overcompensation in the growth of LbL relies on the measurement of specific physico-chemical parameters accounting for the effective charge of the surface, e.g., zeta potential, streaming potential, or surface potential [62,101,104,106,107,108,115,117,118,119,120], which allows evaluation of the oscillation of the surface charge of the multilayer between positive and negative values as alternate layers of polycation and polycation are assembled. Figure 7 shows the changes of the zeta potential, ζ, with the alternate deposition of positive and negatively charged layers of the (PAH-PSS)_n_ multilayer on liposomes with different densities of positive charge (indicated by the percentage of DODAB). The results evidence an oscillation in the zeta potential between values of about +40 mV and −50 mV upon the deposition of polycation and polyanion layers, respectively. It is worth mentioning that even though the results in Figure 7 suggest the existence of a quasi-symmetric overcompensation for the deposition of polyelectrolyte layers bearing opposite charges, the emergence of asymmetrical growth can result in a dependence of the overcompensation level on the specific nature of the last-deposited layer [121]. 

The results in Figure 7 highlight the self-limiting character of the charge inversion process. This means that charge inversion depends almost exclusively on the specific nature of the assembled entities, with the effect of other parameters such as the assembly conditions (e.g., ionic strength or pH) on the overcompensation degree being almost negligible [106,113]. It is commonly accepted that the overcompensation reaches its maximum on the surface of the deposited layers, decaying very fast towards the inner region of the film [107,108]. However, there are no general rules for determining the extension of the overcompensation within the layers, depending on the type of multilayer growth and the layer fuzziness. This leads to a situation in which the overcompensation is propagated along the whole layer for multilayers with non-linear growth [102], whereas in multilayers exhibiting linear growth the overcompensation is mostly restricted to the most external region of the deposited layer [7].

**Figure 8:**
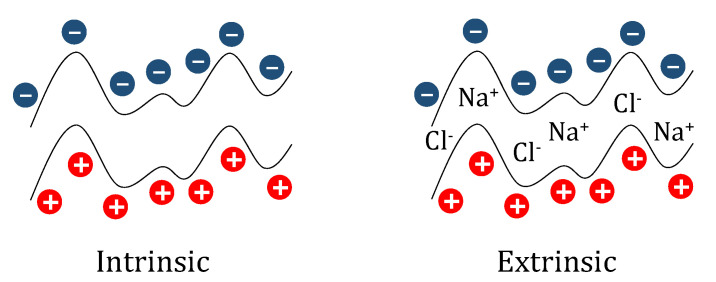
Idealized representation of polyelectrolyte layers and counterions in polyelectrolyte multilayers presenting intrinsic and extrinsic compensation mechanisms. Reprinted from Mateos-Maroto et al. [42], with permission under Open access CC BY 4.0 license, https://creativecommons.org/licenses/by/4.0/ (accessed 20 January 2022).

The above discussion evidences that the overcompensation emerges as a result of the absence of a perfect matching between the charges of the monomers deposited in adjacent layers, which leads to a charge excess on the film. This charge excess must be compensated for to fulfil the electroneutrality boundary condition, guaranteeing the stability of the deposited films [107,122]. Therefore, it is necessary to make an additional contribution that allows counter-balancing of the charge excess to form supramolecular films with zero net charge at the macroscopic scale (beyond the Debye length). This is possible by the incorporation of small ions in the LbL films, thus allowing the compensation of the charge excess emerging from the deposition of the polyelectrolytes [106,109,123,124]. Thus, it is possible to define the compensation of the multilayer charge as a function of the role of small ions in the neutralization process, which leads to two different mechanisms for charge compensation [125,126,127,128]: (i) Intrinsic and (ii) extrinsic. The former charge compensation mechanism is characterized by the perfect matching between the charges deposited in adjacent layers. This means that during multilayer assembly small ions are expelled from the film, leading to the formation of multilayers with stoichiometry of 1:1 between monomers of polycation and polyanion, which in turn results in a high ionic cross-linking between the chains of oppositely charged polyelectrolyte. Therefore, it may be expected that the release of counterions from the multilayered film leads to an important increase in the system entropy, which is favorable for reducing the free energy, and hence the entropy gain becomes the main driving force for the assembly of multilayers having intrinsic compensation. The situation changes for those cases in which the matching between charges of adjacent layers is not enough to ensure the electroneutrality of the multilayered films, and the presence of counterions is necessary for fulfilling the charge balance, resulting in the so-called extrinsic compensation that can drive the formation of multilayers with a broad range of different stochiometries. Furthermore, the retention of counterions within the multilayered films reduces the contribution of the entropy to the assembly process. Extrinsic-like compensation is most common in polyelectrolyte multilayer films [104,105,107,108,109,129], with intrinsic compensation emerging only when highly charged polyelectrolytes are assembled [107,121,130]. Figure 8 displays an idealized picture showing the distribution of polyelectrolyte and counterions in multilayers having different compensation types. 

It should be noted that the compensation mechanism in polyelectrolyte multilayers can be modified by changing the physico-chemical parameters controlling the ionic equilibrium, e.g., ionic strength or pH, which affects the structure and properties of the LbL films [106,107,108]. This is clear from the studies by Schlenoff and Dubas [108], where it was evidenced that the compensation mechanism of (PDADMAC-PSS)_n_ multilayers can be switched from a mainly intrinsic-like compensation at low ionic strength, to an extrinsic one at high ionic strength. Thus, the release of counterions occurring at low ionic strength leads to a favorable entopic contribution to the multilayer assembly process. However, this is not true for multilayers assembled at high ionic strengths, in which a high concentration of counterions remains trapped within the multilayer, and the entropic contribution becomes less important. This leads to two very different energetic landscapes for multilayers following intrinsic and extrinsic compensation mechanisms as a consequence of the different entropic contributions associated with the release of counterions [106,107]. This can be evaluated by introducing the so-called compensation ratio, *R_c_*, evaluating the ratio between the density of positively, $\rho_{monomer}^{+}$, and negatively, $\rho_{monomer}^{-}$, charged monomers in adjacent layers [129]:

(1) $$R_{c}=\frac{\rho_{monomer}^{+}}{\rho_{monomer}^{-}}.$$

Thus, intrinsic compensation may be expected for *R_c_* ≈ 1, whereas extrinsic compensation emerges when *R_c_* assumes values above or below 1, which indicates an excess of cationic or anionic monomers, respectively. For the particular case of (PDADMAC-PSS)_n_ multilayers [106], the *R_c_* values indicate an excess of PDADMAC monomers in relation to PSS one in adjacent layers, with independence of the ionic strength value, which evidences the extrinsic-like character of the compensation process. Furthermore, the increase in the ionic strength pushes *R_c_* from values close to 1 at low values of the ionic strength, i.e., a quasi-intrinsic compensation, to an extrinsic compensation at high ionic strengths (*R_c_* > 1). Furthermore, the compensation presents an asymmetric character, which means that the degree of extrinsic compensation is strongly dependent on the nature of the capping layer [107,131]. Thus, for (PDADMAC-PSS)_n_ multilayers, PDADMAC-capped films present a higher charge excess than those ended in PSS, which suggests a clear extrinsic compensation in the PDADMAC layer, becoming intrinsic for PSS one. The asymmetry of the compensation influences the structure and properties of the layers, which emerge as being strongly dependent on the specific physico-chemical characteristics of the polyelectrolytes. Therefore, if the different compensation in (PDADMAC-PSS)_n_ multilayers is again considered, it may be expected that the presence of counterions in PDADMAC-capped films induces a strong swelling and hydration of these layers, which is associated with a very noticeable roughness. However, the roughness is reduced for PSS layers, which present a more collapsed character. The above picture suggests that the internal balance of charges within the multilayers leads to the presence of extrinsic (polyelectrolyte/counterion pairing) and intrinsic sites (pairing between oppositely charged polyelectrolytes) [107,132]. 

The differences emerging in the compensation mechanism in polyelectrolyte multilayers as a result of the specific chemical nature of the assembled polyelectrolytes, and the assembly conditions, lead to differences in the balance between the enthalpic and entropic contributions driving the assembly [133]. Thus, (PDADMAC-PSS)_n_ multilayers undergo strong exothermic complexation when they are assembled from solutions of low ionic strength, whereas the complexation process emerges as endothermic with the increase in the ionic strength [14], which leads to a very favorable contribution of the entropy to the assembly at low ionic strength. However, the enthalpy and entropy are counteractive for the assembly when high ionic strengths are used. This explains the deconstruction of the films when the multilayers are assembled from solutions of very high ionic strength [134,135].

## 4. Encapsulation in LbL Materials

The exploitation of LbL materials as cargo systems requires a careful examination of the final use of the assembled device [38]. PEMUCs have been commonly fabricated by depositing polyelectrolyte layers onto small drug particles (2–10 μm) or sacrificial cores [136,137]. The former approach is commonly used for the encapsulation of drugs with a reduced solubility in water, allowing the preparation of capsules containing a drug concentration in the range of 70–80% *w/w*. Furthermore, the use of sacrificial particles requires the template removal by a chemical or thermal treatment after the deposition of the layers followed by the encapsulation of the drugs. This requires, in many cases, opening capsules to allow the diffusion of the drugs to the inner region, making it possible to encapsulate the drugs until concentrations in the range of 5–10% *w/w* are reached [138]. Therefore, it is clear that the use of LbL materials with encapsulation purposes requires a careful control of the multilayer shell thickness and chemical composition to ensure the diffusion of the drugs during the loading or release processes.

Moreover, the LbL method offers the possibility to include specific functionalities on the capsule surface, which provides molecular recognition mechanisms for a specific target, and increases the effectiveness of the drugs. In recent years, the exploitation of the LbL method for the fabrication of nanosystems with sizes in the range of 100–200 nm has gained interest, allowing the fabrication of injectable platforms that take advantage of the small size of the fabricated capsules [139,140]. 

It should be noted that the surface charge of the LbL film used as capsules, and their swelling degree, cross-linking density and mechanical properties, play a very important role in the control of the release profiles of the encapsulated compounds [141]. Therefore, careful control of the above parameters is necessary, which can be achieved directly during the assembly process or by a post-treatment once the capsule is assembled [37]. The importance of the increase in the thickness, and the degree of cross-linking, of the capsules in the release profile was demonstrated by Antipov et al. [142]. They studied (PAH-PSS)_n_ multi-layered capsules with encapsulated fluorescein, and found that the thicker the shell, the smaller the permeability of the capsules. Similar conclusions were obtained by studying the release profiles of the ions obtained upon dissolution of calcium oxalate crystals inside a polyelectrolyte shell [143]. 

LbL capsules offer an alternative to the uncontrolled release, commonly mediated by erosion or diffusion, emerging in traditional capsules. Thus, LbL capsules provide strategies for a controlled release taking advantage of the stimuli responsiveness of this type of materials. This allows triggering the release and specific targeting of the encapsulated compounds as a response to a specific external stimulus [144,145], having chemical (pH, solvent or electrochemistry) or physical (temperature, light, ultrasounds, magnetic fields, mechanical deformation) origins, or mediated in an autonomous fashion by living tissue itself [146]. The possibility of controlling the release profile of the encapsulated compounds is essential for the application of LbL capsules in the biomedical field, allowing the optimization of the dosing to obtain a specific therapeutic effect, with the next step towards the fabrication of LbL capsules being the inclusion of a multi-triggered stimuli responsiveness allowing a better mimicking of natural systems [37,146]. 

The application of stimuli is not only useful for a controlled release, and contributes in many cases to the correct distribution of the encapsulated compounds towards a specific target, as was demonstrated by Podgórna and Szczepanowicz [147]. They tested the possibility of driving LbL capsules of poly(l-lysine) and poly(glutamic acid) to their specific target using magnetic fields, which was made possible by including magnetic responsive Fe_3_O_4_ nanoparticles within the capsule core. Light activation with low intensity ultraviolet or near infrared radiations can be also exploited to drive capsules to the target, especially when the interaction of capsules with biological entities is considered [148].

**Figure 9:**
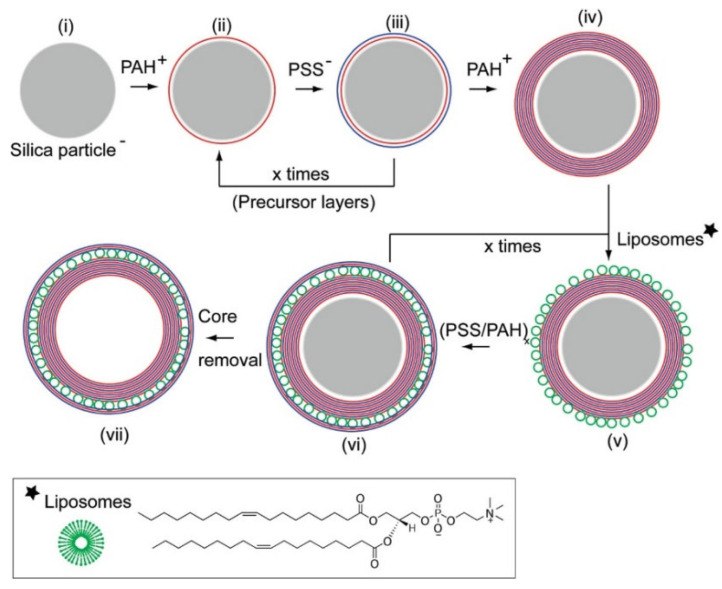
Idealized representation of the fabrication procedure used for obtaining multicapsules. Reprinted with permission from Städler et al. [150], Copyright (2009), American Chemical Society.

In recent years, the design of hierarchical multifunctional capsules containing many compartments (multicapsules) formed by the LbL assembly of several independent subunits has gained interest in different scientific and technological fields, ranging from biomedicine and drug delivery to the fabrication of optical materials, and from the fabrication of biosensors to the design of microreactors [149,150,151]. Figure 9 represents an idealized sketch of the fabrication procedure of multicapsules, which commonly includes the coating of a colloidal template by combining polymer layers and layers of intact vesicles, followed by the removal of the colloidal template.

The fabrication of multicapsules, the so-called capsosomes, has gained attention due to their potential uses as microreactors in chemical synthesis or in the fabrication of artificial cells or organelles with an interest in biomedical applications [149,152]. These hybrid systems combine the advantages of their constituent systems, i.e., traditional LbL systems and liposomes, providing a partial solution to some of their main limitations, e.g., poor mechanical stability of the liposomes. In addition, these multicapsules can include several functionalities, providing interesting avenues for the design of new cargo systems for drug delivery applications [153]. 

It should be stressed that the promising properties of LbL systems as delivery platforms cannot hide their poor stability under specific physiological conditions, which is a very important drawback when the drug accumulation in specific organs and tissues is necessary. A promising alternative for overcoming the instability issues is the coating of the obtained capsules with a layer of a hydrophilic polymers, e.g., polyethylenglycol (PEG), which enhances the stability of the capsules in physiological environments, reducing the fouling phenomena [154,155]. The increase in the number of layers of the LbL shell is also a very common alternative for enhancing capsule stability [156].

### 4.1. Encapsulation Approaches

A very important feature of PEMUCs is their potential use for loading specific cargos into their inner cavity. This allows protecting the cargo materials from inactivation and dissolution, reducing simultaneously their toxicity against cells. There are several approaches for entrapping different cargo materials within PEMUCs. This relies in two types of strategies: (i) Post-loading and (ii) pre-loading [25,157].

#### 4.1.1. Post-Loading

The use of post-loading strategies for the encapsulation of compounds relies on the inclusion of the compounds in the capsules during the final steps of the fabrication procedure, i.e., it is necessary to manufacture a preformed capsule before the loading process, which requires modifying the permeability of the shell by changing some environmental parameter, e.g., temperature, pH, ionic strength, or microenvironmental polarity. This allows the penetration of the compounds to the inner core of the capsule due to the segregation of the polyelectrolyte network and the formation of defects in the shell [158,159]. Once molecules penetrate the capsule shell, the capsules are transferred to their original environment to ensure the retention of the encapsulated molecules. Therefore, it is very important that the modification of the shell permeability does not lead to irreversible changes in the encapsulation matrix to avoid the leaking of the encapsulated compounds from the capsules [160]. This requires choosing the encapsulation methodology depending on the physico-chemical properties of both shell and template to ensure an optimal accumulation of the drug within the inner core of the capsule.

The main drawback of the encapsulation by post-loading approaches is the possible effect associated with the harsh conditions used for template removal, which many times involves the use of toxic solvents, e.g., acid solutions or organic solvents, and hence results in a detrimental effect on the activity of the encapsulated molecules [161]. Furthermore, the dissolution of some templates, e.g., melamine formaldehyde resins, can lead to the formation of residues with an important cytotoxicity effect [137]. It should be noted that post-loading strategies are time consuming, thus limiting their efficiency. They require a high concentration of initial material and the final encapsulation yield is rather limited. Furthermore, the stimuli required for encapsulation and release of the molecules are, in many cases, extreme, which can alter the activity of the encapsulated compounds.

There are several examples involving the encapsulation of drugs in LbL capsules by exploiting post-loading strategies. One of the first examples deals with the inclusion of FITC labelled dextran (FITC: Fluorescein isothiocianate) in (PDADMAC-PSS)_n_ capsules [137]. The results showed that the permeability of the dextran, a high molecular weight polymer (70 kDa), can be modulated through the capsule core by increasing the ionic strength, which leads to an increase in the capsule porosity, enhancing its permeability. Once the dextran was loaded, the capsules were resealed by taking them above the glass transition temperature, forcing shell thickening and densification, which in turn avoided the leakage of the encapsulated compounds. This strategy can be exploited for the encapsulation of materials with a broad range of molecular sizes, from small molecules to larger ones, as was demonstrated by Kozlovskaya et al. [162]. They fabricated capsules of poly(methacrylic acid) and poly(*N*-vinylpyrrolidone) for the encapsulation of a broad range of molecules, and found that the pH responsiveness of the capsules was enough to enable reversible opening and resealing of the capsules, facilitating the encapsulation of small molecules, such as doxorubicin (DOX) or Alexa Fluor 532 dye. However, capsules cannot incorporate species with high molecular weights. Furthermore, the encapsulation of the molecules occurs selectively, with the molecules being distributed within the capsule depending on their charge. Thus, negatively charged molecules are encapsulated within the cavity of the capsules, whereas positively charged molecules are distributed preferentially within the capsule shell. Very recently, Ermakov et al. [163] designed capsules loaded with a photosensitizer for dynamic phototherapy by combining dextran sulfate and poly-(l-arginine), and found that the loading of the drug inside the capsules using a post-loading strategy may be enhanced by increasing the concentration of capsules in the dispersion. Furthermore, they demonstrated that the heating of the capsules after drug encapsulation may be a good strategy to ensure capsule resealing, thus avoiding the leaking of the encapsulated drugs. She et al. [164] also used a post-loading strategy for encapsulating bovine serum albumin labeled with tetramethylrhodamine isothiocyanate in capsules of dextran or alginate and poly-arginine by the incubation of the capsules in a solution containing the labeled proteins for one day at 37 °C, and found a strong dependence of the encapsulation yield on the nature of the shell. Thus, the protein retention in capsules containing alginate is 2.5 times higher than in that encapsulated in capsules containing dextran. The important role of the nature of the shell in the control of the encapsulation process was also evidenced by Ermakov et al. [165]. They showed that the encapsulation of rhodamine B by permeation through the walls of capsules formed by PDADMAC and PSS, and a second formed by poly-arginine and dextran, can be obtained upon thermal treatment, but the emerging thermal treatment is different depending on the shell nature. Thus, rhodamine B is encapsulated in the system formed by PDADMAC and PSS upon thermal treatment at 50 °C for 20 min, whereas for the shell formed by poly-arginine and dextran, greater heating (90 °C) for a longer time (1 h). Is necessary Furthermore, the increase in temperature used for the loading results in very different loading efficacy depending on the specific system. The post-loading by direct incubation in a solution containing the target molecules was also used by Jeannot et al. [166] for the encapsulation of rhodamine 6G in PEMUCs formed by combining PDADMAC and PSS layers.

#### 4.1.2. Pre-Loading

The pre-loading strategies for the encapsulation of active compounds rely on including the target molecules in the PEMUCs during the initial preparation stages, which has been exploited for the encapsulation of poorly water-soluble compounds [137]. The encapsulation during the capsule fabrication procedure leads to a significant reduction in the time involved in the preparation of drug delivery platforms [167].

A very promising option, when pre-loading is considered, is the use of porous colloids, either inorganic or organic, as templates, which allows the incorporation of a high quantity of bioactive compounds within their porous matrix. Among this type of colloid, CaCO_3_ particles are probably the most commonly used particles as templates for the fabrication of PEMUCs, particularly due to their biofriendly character [168]. This is in part due to the possible fabrication with a narrow size distribution, generally in the 2–4 μm range. Furthermore, they present a large specific surface area, which allows the encapsulation of large quantities of target molecules [25]. The use of CaCO_3_ allows encapsulation directly during their synthesis by co-precipitation, or once the colloids are obtained by diffusion and incorporation into the pores [168,169]. Therefore, encapsulation can be performed in preformed capsules or carried out simultaneously during the template preparation [170]. It should be noted that the final encapsulation yield obtained using pre-loading strategies depends on the specific nature of both capsules and encapsulated molecules [25,171]. The final capsules are generally obtained upon removal the template by a mild treatment. However, this can limit in many cases the materials that can be encapsulated. Thus, CaCO_3_ particles, which are commonly removed upon exposure to slightly acids aqueous solutions of ethylene-diamine-tetraacetic acid, cannot be used for the encapsulation of pH responsive molecules, or molecules containing di- or trivalent metal ions. Some of the molecules pre-loaded in capsules obtained using CaCO_3_ particles as templates are dextran, α-lactoalbumin, lysozyme, horseradish peroxidase, glucose oxidase, catalase, ovalbumin, bovine serum albumin, α-chemotrypsin, insulin, DNA, and pronase [172]. Balabushevich et al. [170] demonstrated that the use of a pre-loading approach for including proteins within sacrificial CaCO_3_ allows reaching high protein concentrations within the final PEMUCs. However, the concentration of encapsulated compounds appears strongly dependent on the methodology used for the pre-loading. Thus, the encapsulated amount is higher when co-precipitation methods are used than when the molecules are encapsulated by simple adsorption on the colloidal template. The use of mesoporous silica particles as templates is a possible alternative to calcium carbonate ones [173,174]. However, silica presents a very important drawback related to its dissolution process, which requires the use of harsh conditions (hydrofluoric acid solutions). However, this is not a problem for its application in encapsulation of different molecules, including urease, DNA, and catalase [175].

The use of reticulate hydrogel beads (agarose, hyaluronic acid, alginate, chitosan, or dextran) as templated for the fabrication of PEMUCs, instead of porous inorganic colloids, has gained interest in recent years, especially because they offer very interesting responsiveness against different stimuli (pH, T, ionic strength), which allows a controlled loading and release of active molecules [152,176,177]. Hydrogel beads have been commonly exploited for the encapsulation of water-soluble compounds taking advantage of the high water content of the hydrogel matrix. In particular, hydrogel beads have been exploited for the encapsulation of different peptides or proteins ensuring the protection of their bioactivity [178].

**Figure 10:**
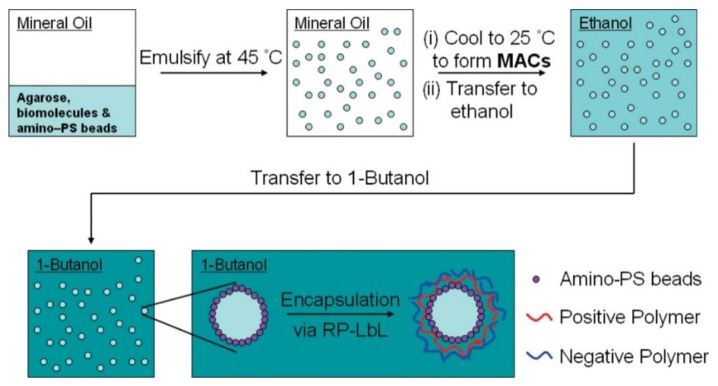
Sketch representing a general perspective of the encapsulation using the RP-LbL approach. Reprinted from Mak al. [179], Copyright (2009), with permission from American Chemical Society.

The use of reverse-phase LbL encapsulation (RP-LbL) is also a very interesting methodology for the encapsulation of drugs with a high solubility in water [179], e.g., the enzymatic pair formed by horseradish peroxidase (HRP) and glucose oxidase (GOD) [180]. This is enabled by including the molecules to be encapsulated in water droplets, whereas the polymer is included within the continuous organic phase. Beyer et al. [181] demonstrated that RP-LbL can be exploited for the encapsulation of a broad range of water-soluble molecules, including high molecular weight proteins (e.g., bovine serum albumin, 65 kDa), low molecular weight organic substances (e.g., glucose, vitamin C, citric acid sodium salt), and inorganic salts (e.g., sodium chloride). This was enabled by the use of non-ionized polyelectrolytes with high solubility in organic medium, which can be obtained following a protonation process. The advantage of the use of this approach is associated with the possibility of obtaining capsules that can be lyophilized under mild conditions, allowing their redispersion in an aqueous environment. This simplifies the encapsulation of water-soluble drugs. Pan et al. [182] demonstrated that the application range of capsules obtained by RP-LbL approaches can be expanded, e.g., for the fabrication of edible capsules [183], by controlling different parameters during the assembly of the capsules. Thus, the change in the polyelectrolyte concentration, number of formed layers, nature of the polyelectrolyte, and water fraction used during the assembly allows modulating the thickness of the LbL shell in addition to its density, adhesive properties, and fuzziness. A very interesting application of the capsules obtained by RP-LbL is their use as micro-reactors for performing reactions in controlled environments. In particular, Mak et al. [184] demonstrated that this type of capsule may be exploited for performing a high number of individual polymerase chain reactions (PCR). This offers a good alternative for the designing of new diagnostic strategies. Figure 10 presents a sketch in which the preparation of LbL capsules using a RP-LbL methodology is depicted.

The pre-loading encapsulation can be also exploited by the direct coating of crystalline templates formed by the molecules to be encapsulated, which is very useful for drugs with low solubility under the conditions used for the encapsulation [137]. 

The encapsulation of hydrophobic drugs requires the design of complex strategies guaranteeing their trapping in the LbL structure, which can be achieved using templates including well-defined hydrophobic regions, e.g., oil droplets in emulsions, micelles, vesicles, or liposomes, or simply hydrophobically modified polyelectrolytes [185,186]. The latter were exploited for paclitaxel encapsulation in capsules formed by hydrophobic modified hyaluronic acid and quaternized chitosan [187]. The use of this type of capsule offers a controlled release of the encapsulated drug, which can be more finely controlled using hyaluronidase, thus allowing a controlled opening of the shell [188]. Emulsion droplets, which act as an encapsulation/solubilisation environment, are widely used as templates for the fabrication of PEMUCs, allowing the use of pre-loading strategies for the encapsulation of different molecules [186], e.g., anticancer drugs such as 5-fluorouracil and doxorubicin, DNA, RNA, or different oligonucleotides [189,190]. The strong development of the encapsulation in emulsion droplets occurs due to the high concentration of drugs that can be included within the oil droplets. Furthermore, this type of carrier allows the enhancement of some properties of the encapsulated materials, e.g., their permeability through cellular membranes or susceptibility to chemical or enzymatic degradation [191].

### 4.2. Controlling the Release of Encapsulated Molecules

**Figure 11:**
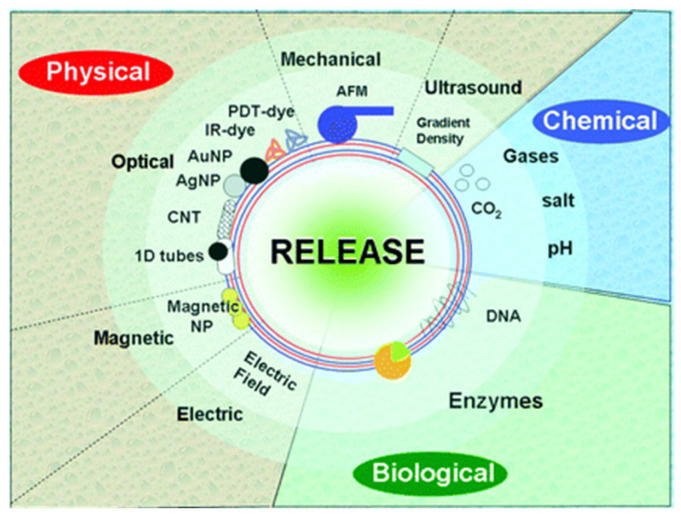
Scheme of different type of stimuli that can be exploited for encapsulation and release of encapsulated compounds from LbL materials. Reproduced from Skirtach at al. [37], Copyright (2011), with permission from The Royal Society of Chemistry.

A successful release of the encapsulated drug is essential for the design of suitable platforms for biomedical applications, ensuring an effective action in a controlled manner. This is possible by exploiting the stimuli responsiveness of the capsules, which requires consideration of the specific physico-chemical properties of the encapsulated compounds and capsule shell. The origin of the stimuli contributing to the release of encapsulated compounds can be very different. Thus, there are different chemical or biological stimuli that emerge directly as a result of the environmental conditions encountered internally, e.g., pH, ionic strength, polarity, temperature, enzymes, or receptor recognition. Conversely, different external stimuli associated with the interaction of the capsules with externally applied fields, e.g., magnetic field, ultrasound, light irradiation, or mechanical stress, can also be exploited for triggering the release of encapsulated compounds. Therefore, the drug release can be triggered following different strategies, with the choice of the more suitable approach depending on the type of drug, the exposure area, and the purpose of the delivery. Figure 11 shows a scheme displaying the different type of stimuli that can be exploited for the controlled release of encapsulated compounds from PEMUCs. 

#### 4.2.1. Environmental Induced Release

The environmental conditions, i.e., pH, ionic strength, solvent nature, etc., are frequently used for controlling the interaction balance during the assembly of LbL materials to obtain supramolecular systems with a defined structure and physico-chemical properties [14]. However, the change in the environmental conditions can be also exploited in preformed PEMUCs for modulating the release profiles of encapsulated compounds. Thus, ionic strength changes allow a reversible modification of the polyelectrolyte shell as a result of the induced osmotic stress. This has been demonstrated for (PDADMAC-PSS)_n_ and (PAH-PSS)_n_ multilayers, which undergo a strong swelling upon the decrease in the ionic strength of the environment. This favors the release of counterions from the inner region of the shell, and can be useful for triggering the release of encapsulated compounds from PEMUCs [107,118].

Moreover, the change in the ionic strength can be also exploited for a controlled erosion of the LbL shell by inducing desorption of polyelectrolyte layers favoring the release of the encapsulated compounds. This has been probed in (PDADMAC-PAA)_n_ capsules, where an increase in the ionic strength above a threshold concentration of 600 mM leads to the disassembly of PAA layers, helping the release of the encapsulated compounds [192]. A similar result was found for capsules formed by hydrogen bonds [193].

The modification of the environmental pH also plays a very important role in the control of the interaction balance within the multilayer, and can be exploited for triggering the loading and release of compounds from PEMUCs. However, in contrast to the case of the ionic strength, the pH changes can be only used for controlling the release profiles of capsules containing weak polyelectrolytes, i.e., polyelectrolytes having an ionization degree that depends on the specific environmental pH. Thus, when the pH is modified in the vicinity of the pK_a_ value, it is possible to modulate the protonation/deprotonation equilibrium of the polyelectrolytes, which leads to a swelling of the multilayers due to the change in the ionization degree of the polyelectrolyte films forming the multilayer [194].

Müller et al. [195] demonstrated that pH changes can be exploited for the reversible loading and release of positively charged proteins from multilayers formed by poly(ethylenimine) and PAA. Thus, at neutral pH it is possible to load the positively charged proteins in the capsules due to their electrostatic interactions with the negatively charged PAA layers, whereas the decrease in the pH leads to the protonation of PAA monomers, inducing the release of the trapped proteins. The controlled release by pH changes is key for applications in physiological media that can present very different pH, e.g., (pH 1–2), intestine (pH 8.4), or endosome (pH 6.0–6.5) [13]. Therefore, the pH changes can be exploited for targeting in different parts of the body [196]. This plays a central role for a specific drug release in carcinogenic tissues due to the differences between their pH (pH < 6.8) and that of healthy tissues (pH around 7.4) [144]. Therefore, it may be expected that the design of delivery platforms with strong responsiveness to pH changes within a defined pH range can contribute to the enhancement of the efficacy of different biomedical treatments. The power of the pH to modulate the release profile in carcinogenic tissues of doxorubicin from PEMUCs formed by chitosan and hyaluronic acid was evidenced by Zhao and Liu [197], who showed that, for capsules formed by such specific polyelectrolyte pairs, a faster release of the encapsulated drug in carcinogenic tissues than in healthy ones is possible. This offers important advantages for modulating the release of doxorubicin in tumors from other PEMUCs [198,199]. It should be noted that the pH effect on the modulation of the release profile is strongly dependent on the specific nature of the multi-layered structure, as was demonstrated by Han et al. [200]. They explored the pH-induced release of a model drug encapsulated (coumarin) in two different pH responsive multilayers formed by the assembly of an amphiphilic block copolymer of polystyrene and PAA with amino functionalized graphene oxide and branched poly(ethyleneimine), and found a faster release from multilayers containing the latter than from those containing graphene oxide, which was explained considering that the ionization degree depends strongly on the specific chemical characteristic of the assembled species. 

The specific nature of the solvent, and in particular its polarity, can also be exploited for modulating the properties of the capsule shell. In particular, organic solvents allow modulating the permeability of capsules by inducing changes in the shell porosity, which can be rationalized considering that the solvent polarity alters the balance of interactions within the multilayer, and consequently its structure [175,201]. Thus, (PAH-PSS)_n_ are insoluble after their fabrication in aqueous medium. However, they become soluble in ethanol, which allows controlling their permeability by changing the ethanol content in the solution. This has been exploited for the controlled loading and release of urease from (PAH-PSS)_n_ capsules via a reversible opening and closing of their shell by adding ethanol and water, respectively. However, the addition of ethanol leads to a partial inactivation of the enzyme [175].

#### 4.2.2. Physically Induced Release

There are different external physical stimuli that can contribute to trigger the release of encapsulated compounds from PEMUCs. Temperature-triggered release is probably the most commonly used external physical stimuli, allowing the control of the layer organization and hydration degree, which can be exploited for modulating the capsule permeability and consequently the release profiles [202]. This is enabled by incorporating elements undergoing a reversible hydrophilic-hydrophobic phase transition as response to temperature changes. Thus, the temperature changes provide a suitable strategy for altering the properties of specific materials such as the poly(*N*-isopropylacrilamide) hydrogels. These colloidal systems undergo a swelling-shrinking reversible transition at a temperature close to the physiological one [203], which can be exploited for loading and controlled release of drugs from the hydrogel matrix [204]. Zhu et al. [205] fabricated a LbL material by including thermoresponsive micelles of poly(*N*-vinylpyrrolidone)-block-poly(*N*-isopropylacrylamide) loaded with doxorubicin in a LbL material, and found that the increase in the temperature above the physiological one (around 37 °C) allows trapping the drug within the LbL material, whereas the decrease in the temperature to values of about 20 °C allows triggering a fast release of the encapsulated doxorubicin. Thus, the release and retention of the drug can be triggered cyclically on demand several times (up to 15 cycles) as a response to temperature changes. Temperature-triggered release can be also used in classical multilayers such as those formed for PDADMAC and PSS. Zhuo et al. [206] demonstrated that temperature changes can stimulate the release of dexamethasone from (PDADMAC-PSS)_n_ capsules in such a way that is strongly dependent on the number of layers, ionic strength, temperature, and nature of the outermost layer. 

Laser light activation can be used for inducing a local heating of the capsule, which triggers the release of the encapsulated compounds [207]. This requires introducing optically active components in the LbL shell, which is commonly achieved by embedding metal nanoparticles, playing a very important role in intracellular release [208], multi-substance delivery [209], or endosomal escape [210]. The mechanism of the laser light activation relies on the heating of the particles by light irradiation, which can induce the rupture of the shell or the modification of its permeability [31,211]. Thus, the use of near infrared radiation allows triggering the release of doxorubicin from multilayers formed with poly(l-lysine), bovine serum albumin, and a thermoresponsive gelatin hydrogel in which gold nanoparticles were embedded [212]. Similar results can be obtained by the embedding of silver nanoparticles within the capsule shell [208]. It should be noted that the choice of near infrared radiation for triggering the release of compounds from LbL capsules is associated with their limited impact for cells and tissues [213]. The use of pulsed lasers can be also a good alternative for the release of different compounds from capsules as a result of the formation of pores in the shell, as was demonstrated by Radt et al. [214] and Skirtach et al. [208] in the release of lysozyme from (PAH-PSS)_n_ capsules and dextran from (PDADMAC-PSS)_n_ capsules into hippocampal neuron cells, respectively. Gupta and Sivakumar [215] demonstrated the power of light-triggered release as a tool for the treatment of gangliosidosis. For this purpose, they studied the internalization into different type of cells of (PAH-PSS)_n_ PEMUCs with embedded gold nanoparticles and loaded with β-galactoside, which was easily released for the heating resulting from the exposure to near infrared radiation (with a wavelength of 980 nm and a power of 1 W). Similarly, Kurapati and Raichur [216] designed capsules formed from alternate layers of PAH and graphene oxide, which were loaded by doxorubicin. These capsules can release the encapsulated drug upon laser irradiation, with the velocity of the release being strongly dependent on the time of irradiation. This can be explained considering that the irradiation of the capsules with near infrared radiation results in the formation of a hole in the capsule which grows as a function of the progress of the heating process.

Light irradiation can be also exploited for triggering the release of encapsulated compounds by the addition of photosensitive groups, e.g., benzyl, 3-methoxybenzyl, 3,5-dimethoxybenzyl, or 2-nitrobenzyl, to the capsule shell [217]. This was used by Wang et al. [218] for triggering by visible light irradiation the in vitro release of Hypocrellin B from capsules of chitosan and alginate in human cancer breast cells.

Ultrasounds can also be exploited for triggering the release of encapsulated compounds from LbL capsules. However, their exploitation requires inclusion of materials that confer the sensitivity to this type of radiation. Shchukin et al. [219] demonstrated the possibilities of the use of ultrasound irradiation for the release of FITC-dextran from (PAH-PSS)_n_ capsules by including Fe_3_O_4_ particles. Kolesnikova et al. [220] obtained similar results using ZnO nanoparticles to ensure the capsule responsiveness to ultrasound radiation. Despite the interest in the use ultrasound for triggering the release of encapsulated compounds, its in vivo uses are limited due to the difficulties associated with the use of an ultrasound acceptable intensity suitable for the release in medical applications. Stavarache and Paniwnyk [221] showed that the release profile can be modulated depending on the position of the polyelectrolyte shell where ultrasound sensitive particles are placed. Thus, when particles are placed close to the central core of (PAH-PSS)_n_ capsules, the drug release occurs following a sustained profile, whereas a burst release mediated for the capsule breakage is found when particles are placed close to the capsule surface. Microwave radiation can be also used as an alternative to trigger the release process as demonstrated by Borodina et al. [29]. They recently proposed the use of nanodiamonds embedded within a polyelectrolyte shell as a tool for triggering the release of the encapsulated compounds. Thus, the thermal stress induced in the multilayer upon microwave irradiation leads to nanodiamond detonation, which heats the surrounding polyelectrolyte layers, contributing to the opening of the microcapsules and enabling the release of the encapsulated molecules.

The application of magnetic fields can be used for triggering the release from LbL capsules, as shown the studies by Lu et al. [222]. They used alternate electromagnetic fields to induce the oscillation of gold-coated cobalt nanoparticles loaded in (PAH-PSS)_n_ capsules, inducing a disturbance in the polyelectrolyte shell, which modifies its permeability. However, the application of magnetic fields can induce a strong temperature increase, which limits their application in real systems due to their potential effects on the stability of the encapsulated molecules, and their undesirable impact on target tissues and cells [223]. This can be solved by using low frequency magnetic fields, as demonstrated Burmistrov et al. [224]. They embedded single domain maghemite nanoparticles integrated within the shell of (PAH-PSS)_n_ shells, which can act as magneto-mechanical actuators that modify the permeability of the capsules without any significant modification of the system temperature, allowing a sustained release of the encapsulated compounds by increasing the exposure time [225]. 

#### 4.2.3. Redox Induced Release

Redox processes are also an excellent approach for modulating the release of encapsulated drugs from LbL capsules [144,226], taking advantage of the reductive environment provided for the intracellular region. Thus, redox processes can modify the potentials, thus breaking the charge balance, which in turn leads to an increase in the osmotic pressure. This induces a deformation of the capsule, which can lead to its disintegration. The use of this approach for triggering the release is possible when LbL capsules are fabricated in such a way that they maintain their stability during their transport along the extracellular fluid, and they undergo a destabilization process once they penetrate into the interior cellular, which triggers the release of the encapsulated compounds [227]. This type of release allows enhancing the cytotoxicity of doxorubicin against colon cancer cells by its encapsulation in capsules formed by poly(methacrylic acid) and poly(vinylpyrrolidone) chemically cross-linked by using a redox active bisazide linker [228]. The use of redox controlled release has also been explored for the release of FITC-bovine serum albumin from capsules composed of cysteamine conjugated chitosan and dextran sulphate [229]. 

#### 4.2.4. Biochemical Induced Release

The release of encapsulated compounds can be triggered as a result of biochemical reactions originated for the specific physiological conditions of the systems or induced by enzymatic activity. Thus, the physiological conditions, mainly pH or salinity, can induce the release of the encapsulated compounds without the application of any external stimulus [230]. This may occur as a result of two different types of processes: (a) Capsule erosion [231] or (b) breaking of the bonds allowing the binding of the drug to the capsule [232].

The release triggered by enzymatic action is used frequently in capsules containing some biofunctional materials, e.g., polypeptides or polysaccharides, which can be suitable substrates for specific enzymes [233]. Thus, drugs encapsulated in capsules composed by poly(lactic acid) and poly(ethylenimine) can be released by α-chymotrypsin [234], whereas chitonase can be used for triggering the release of drugs from capsules formed by the combination of dextran sulfate and chitosan [235]. Proteases can triggering the in vivo release of drugs encapsulated in capsules of poly(l-arginine) and dextran sulphate [236]. Similarly, Borodina et al. [237] enabled triggering of the release of encapsulated compounds from capsules formed by poly(l-arginine) and poly(l-aspartic acid), with the release being spanned in time-scales ranging from a few seconds to several hours, or even days, depending on the nature of the protease involved.

## 5. Layer-by-Layer Capsules for Antibiotic Release

The design of strategies to control the release kinetics of antibiotics is essential for effective therapy, and, in particular, to reduce sub-inhibitory levels, which can induce resistance phenomena [238]. This can be solved, at least in part, by exploiting the potential of LbL PEMUCs. Al Thaher [239] proposed that the use of LbL (PAH-PSS)_n_ capsules can be a good alternative for an optimal release of the antibiotic gentamicin. The capsules undergo a sustained release of the antibiotic (during 2 weeks) triggered by acidic conditions. This is important because it can be exploited for antibiotic release under acidosis conditions induced by the microbial infection, as was demonstrated by Craig et al. [240]. They used (PLL-HA)_n_ capsules loaded with specific antibiotics (vancomycin and *polyhexamethylene biguanide*) against *Pseudomonas aeruginosa* and found that the acidic conditions originated for the action of the protease segregated for the bacteria leads to a degradation of the polyelectrolyte shell, allowing the release of the drug. The release in the acidic conditions induced as a result of the bacterial infection was also probed by Zhuk et al. [241] as a very promising tool for exploiting the therapeutic action of several antibiotics (tobromycin, gentamicin, and polymyxin B) against *Staphylococcus epidermidis* and *Escherichia coli*.

Pawlak et al. [242] tried to fight against kamayicin-resistant *Escherichia coli* using capsules formed by the alternate assembly of two dextran derived polymers, the first cationic (diethylaminoethyl-dextran hydrochloride) and the second anionic (dextran sulphate), and loaded with kamayicin. They found that the encapsulation overcame the resistance of bacteria, resulting in an inhibitory effect of the bacterial growth. This inhibitory effect is enhanced with the increase in the biopolymer layers, and the removal of the template used for the capsules’ assembly (calcium carbonate), which is associated with an improved adhesion to the bacterial membrane due to the higher flexibility of the capsules. Table 1 summarizes some examples of LbL capsules used for the release of antibiotics.

## 6. Layer-by-Layer Capsules for Anticancer Therapy

The encapsulation and delivery of anticancer drugs is extremely difficult due to the impact of different parameters, including their poor permeability in solid tumors, the difficulties associated with reaching the tumor, and the strong side effects associated with the systemic administration of drugs. The use of LbL capsules for encapsulating antitumoral drugs with a broad spectrum of activities provides important avenues for a more efficient antitumoral drug administration [144,243].

Jing et al. [244] proposed the use of paclitaxel loaded multilayered capsules formed by alternate layers of hyaluronic acid modified with β-cyclodextrin and poly-(l-lysine) as a therapeutic tool against breast cancer cells (MDA-MB-231). These capsules remain stable under physiological conditions, undergoing a sustained release of the encapsulated drug (a complete release of the encapsulated drug was reached after 5 days). Furthermore, capsules can bind specifically to the tumoral cells, even they are not internalized, through the CD44 receptor sensitive to hyaluronic acid, which is overexpressed, resulting in a strong reduction in cell viability (about 80% of mortality after 3 days of exposure). The encapsulation of the paclitaxel enhances its anticancer power due to its direct and continuous release from the capsules to the tumoral cell. This is possible because the hyaluronidase enzyme secreted by breast cancer cells can degrade the hyaluronic acid, facilitating the drug release.

Vergaro et al. [245] explored the behavior of different polyelectrolyte multilayered capsules loaded with *cisplatin* against different lines of tumoral cells, including MCF-7 (breast cancer), SKOV-3 (ovarian cancer), HeLa (cervical cancer), and CACO-2 (human epithelial colorectal adenocarcinoma). This was considered a good strategy for an enhanced bioavailability of the encapsulated drug, with the internalization of the capsules being in all cases above 50%, and achieving the maximum uptake value for breast cancer cells (75% of the capsules). Furthermore, the release of the *cisplatin* was found to be slower than the time scale involved in the internalization of the capsules, providing potential therapeutic uses of the fabricated capsules. However, encapsulation of *cisplatin* does not induce any significant change in their cytotoxicity with respect to the free form, even though the internalization of the capsules is clearly enhanced. An enhanced internalization was also found for doxorubicin in the study by Shen et al. [246]. They reported that the cellular uptake and the cytotoxicity of doxorubicin is significantly enhanced upon encapsulation on LbL capsules obtained by deposition of chitosan and alginate layers on bovine serum albumin hydrogels. Thus, the capsules ensure a high drug accumulation in MCF-7/ADR tumoral cells, providing a prolonged retention in tumor sites, which contributes to reduction in the tumor growth. Similar results were reported by Trushina et al. [247] using capsules formed by dextran sulphate and poly(l-arginine). Furthermore, it was found that the effect of encapsulated doxorubicin is enhanced when the structure of the capsules is suitable for ensuring a sustained release. Other LbL PEMUCS with a good ability for a long-term retention of doxorubicin are those formed by combining tannic acid and poly(*N*-vinylpyrrolidone) [248]. (PAH-PSS)_n_ multilayers have also been used for the fabrication of capsules for doxorubicin encapsulation. These capsules offer the most suitable conditions for drug release at low pH and relatively high ionic strength. Furthermore, both pH and ionic strength allows modulating the release of the encapsulated drug [249]. 

The encapsulation of *cisplatin* was also tested from (PLL-PGA)_n_ capsules, resulting in the formation of capsules with the anticancer drug embedded within the polyelectrolyte shell. The drug release was shown to be possible under acidic or reductive conditions; this finding is interesting for the release of the drug in the cytoplasm of carcinogen cells. This induces a stronger cytotoxic effect upon capsule internalization for colon cancer cells CT-26 than the free *cisplatin*, which is the result of the enhanced internalization of the drug as a result of its encapsulation within PEMUCs [250].

Capsules formed by the anticancer protein drug protamine and heparin loaded with doxorubicin were used as platforms for antitumoral therapy in MCF-7 breast cancer cells. This type of capsule was internalized by cells where they were eroded as result of the pH change. This allows the release of the encapsulated drug, resulting in cell death. Furthermore, the encapsulation allows enhancing the bioavailability of the encapsulated drug [251]. LbL capsules of poly(methacrylic acid) and poly(*N*-vinylpyrrolidone) were used for the encapsulation of the potent anticancer drug 7-(benzylamino)-3,4-dihydro-pyrrolo[4,3,2-de]quinolin-8(1H)-one (iminoquinone). This type of capsule presents an in vitro sustained drug release as a result of the differences in the redox potential between the intra- and extra-cellular environments. This leads to a fast release of the encapsulated drug (80% of the encapsulated concentration after 24 h) upon penetration into the cells as a result of the reducing environment provided for the presence of glutathione. Furthermore, the encapsulation enhances the cellular uptake of the drug in relation to the free form, reducing the concentration required for inducing a therapeutic effect in HepG2 and Huh7 hepatocellular carcinoma cells, with a minimal impact on healthy tissues. In addition, the release of the drug encapsulated within the LbL capsules results in a significant alteration of the metabolism pathways involved in the proliferation and growth of HepG2 liver cancer cells and Huh7 hepatocellular carcinoma cells. Thus, the encapsulation amplifies the potency of the drug to downregulate the expression of oncogenic proteins, and upregulate the expression of tumor suppressor and cell proliferation suppressor proteins, indicating the good selectivity of the capsules in anticancer therapy [252].

Kittitheeranun et al. [253] exploited capsules formed by the classical polyelectrolyte combination of PDADMAC and PSS for encapsulating curcumin, with the drug-loading efficiency and release profile being mediated for the hydrophobic interactions between the drug and the capsule shell. The optimal release of the anticancer drug was obtained under acidic conditions, which can assist with a suitable curcumin release into tumoral cells, as evidenced by the inhibition of the growth of HeLa cells and their negligible impact on healthy cell viability (human fibroblast cells).

LbL capsules are not limited to the encapsulation of single drugs, as evidenced by the work by Sharma et al. [254]. They combined PAH and poly(methacrylic acid) with embedded gold nanoparticles for encapsulating doxorubicin and nimbin, which can be released upon photothermal activation using near infrared irradiation. It should be noted that the encapsulation of the drug occurred in a selective way, with the hydrophilic drug (doxorubicin) being encapsulated within the inner cavity of the capsules, and the hydrophobic one within the porous polymer shell. Thus, the release of the drug from the capsules into THP-1 cancer cells results in an excellent antitumoral activity. The encapsulation of more than one drug was also exploited by Ramasamy et al. [255] for simultaneously encapsulating doxorubicin and mitoxantrone. They prepared multilayered capsules poly-(l-lysine) and poly(ethylene glycol)-*block*-poly(l-aspartic acid) on liposomes, which offer many functional groups and compartments for ensuring an optimal encapsulation of the anticancer drugs. Furthermore, this type of capsule offers a different release profile for each drug, which is modulated for the acidic conditions emerging in cancer cells. Furthermore, the encapsulation of the drugs extends the time of the systemic circulation of the drug, reducing the elimination rates. The pH responsiveness of capsules formed by chitosan and poly(ethylene glycol dimethacrylate-co-methacrylic acid) was also exploited by Kazemi-Andalib et al. [256] for the controlled release of the combination of anticancer drugs formed by curcumin and gemcitabine. Thus, the acidic conditions encountered by the PEMUCs, following uptake for colorectal carcinoma cells, trigger the drug release, inducing a high cytotoxicity, which emerges in a dose-dependent manner.

One of the most important drawbacks in the biomedical application of PEMUCs is associated with their biocompatibility, which can significantly affect the capsule internalization. This can be partially overcome by modifying the external surface by the deposition of a lipid bilayer, as was demonstrated by Shao et al. [257]. They fabricated LbL capsules loaded with doxorubicin by the alternate assembly of chitosan, alginate, and gold nanorod layers. These capsules can undergo a remotely photoactive drug release upon irradiation with near infrared light, which induces a fast death of the tumoral cell, inhibiting the tumor growth without side effects on healthy tissues. Table 2 summarizes some examples of LbL capsules used for the release of antitumoral drugs.

## 7. Layer-by-Layer Capsules for Gene Delivery

Gene delivery relies on the introduction of exogenous genes into host cells for therapeutic purposes. This is enabled by the translation of information contained in the genes for the production of functional proteins exploiting the machinery of the cells [258,259,260]. Thus, gene delivery can be exploited for the treatment of different diseases, and also has applications in vaccination [259,261]. However, the success of gene delivery therapies requires technologies that allow an effective introduction of the genetic information using viral or non-viral vectors. The latter is the preferred option for avoiding the high immunogenicity of viral vectors [259,262,263], with LbL capsules being suitable alternatives for the delivery of genetic material [264]. Thus, the LbL shell provides protection against nucleases and other aggressive factors, minimizing any potential degradation of the genetic material during its transport into the nucleus of the cell. The polyelectrolyte character of nucleic acids enables their inclusion in LbL capsules as a part of the shell combined with a cationic polyelectrolyte [265,266], or in the inner cavity of the capsule [267].

Santos et al. [268] proposed the fabrication of non-viral gene vectors based on the use of LbL capsules. They efficiently encapsulated DNA plasmids in LbL capsules formed by (PAH-PSS)_n_ multilayers and (dextran sulphate-poly-(l-arginine))_n_, and found a very efficient transfection into NIH 3T3 fibroblasts for both types of vector. This was significantly improved by adding iron oxide particles within the capsule. However, the biodegradability of the polymers forming the capsules is a very important control parameter modulating the gene expression level. The encapsulation of a DNA plasmid in LbL capsules was also shown as a suitable alternative for the preparation of vaccines against swine fever, as in vivo tested by Selina et al. [269]. Reibetanz et al. [270] designed LbL capsules formed for the alternate deposition of protamine and dextran sulphate incorporating DNA plasmids as antitumoral therapy. These capsules were taken up for HEK 293 cells through a non-receptor-mediated endocytotic pathway, resulting in a defoliation-induced release of the plasmids once they penetrated the cells, as evidenced by the expression of the proteins, enhanced green fluorescence protein (pEGFP-C1), and a red fluorescence protein (pDsRed1-N1), for which the plasmids encoded. Furthermore, the release mechanism was found to be enhanced when protamine appeared as the last layer.

Tarakanchikova et al. [271] probed the efficiency of LbL capsules of dextran and poly-l-arginine, with sizes ranging from several nanometers to a few micrometers for the intracellular delivery of messenger RNA (mRNA) and small interfering RNA (siRNA). They found that this type of non-viral vector allows the packing, and subsequent transfection, of both types of genetic material into human mesenchymal stem cells (hMSCs). No significant differences were found in relation to the cellular uptake of the capsules as a result of their size. However, microcapsules have the highest efficiency of siRNA transfection. Furthermore, the transfection efficiency of the genetic material encapsulated within LbL capsules was reported to be almost two-fold higher than that found in commercially available formulations. Therefore, LbL capsules may be considered to be a universal vector for nucleic acid transfection into primary human cells.

Kakran et al. [272] demonstrated that the encapsulation of mRNA within LbL capsules of poly-(l-arginine) and dextran sulphate prevents the degradation of the nucleic acid as a result of the action of the RNAse inside cells. However, the efficiency of the transfection was rather low because the used capsules do not allow the release of the nucleic acid in the right place.

Xie et al. [273] demonstrated the transfection ability on different cellular lines of polyplexes formed by the alternate assembly of DNA with different polycations (poly(ethyleneimine) and poly(amino pentanol)), finding that the transfection is enhanced and the cytotoxicity reduced when lipoplexes with poly(amino pentanol) are used. Polyelectrolyte capsules with siRNA included within a polyelectrolyte shell formed by the alternate deposition of hyaluronic acid and poly(ethyleneimine) were tested for inducing the knockdown of the ICAM-1 gene in human umbilical vein cell line EA.hy926. The efficiency of this gene delivery vector was found to be higher as the number of siRNA layers included in the polyelectrolyte shell increased, even for systems in which the concentration of siRNA is comparable [274]. Table 3 summarizes some examples of LbL capsules used for gene therapy and the type of genetic material contained for such capsules.

It should be noted that the transfection capacity of non-viral LbL capsules in gene delivery depends on several parameters, including the strategy used for loading the genetic material, the incorporation of additives together with the genetic material, and the variation in the incubation conditions. The optimization of these three factors allows a highly efficient transfection [275].

## 8. Layer-by-Layer Capsules for Diabetes

Diabetes is a metabolic disease associated with an abnormal increase in the levels of glucose in blood, which leads to several health complications [276]. As a result, the regulation of the level of insulin and its delivery to the liver to reduce the risks of hypoglycemia and hyperinsulinemia are challenges for diabetes therapy [277]. In addition, this therapy requires the cell barrier to be overcome to ensure the distribution of insulin along the blood stream, making it necessary to design carriers with a high drug loading. Zhang et al. [278] attempted to address this important health challenge using liposomes decorated with a LbL shell formed by the combination of chitosan and insulin layers, in which the increase in the chitosan was essential for an enhanced loading of insulin. This is due to the condensation of more insulin molecules as a result of the increase in the available charges. The release of the insulin is mediated by the erosion of the capsules under acidic conditions, in which both chitosan and insulin are positively charged and undergo a strong electrostatic repulsion. Conversely, the release of insulin is slowed at neutral or slightly basic pH. The obtained capsules allow the retention of the insulin bioactivity and crossing of the epithelial barriers of 3T3 L1-MBX adipocytes, resulting in a good response upon glucose exposure. Capsules formed by alternately deposited poly(vinyl alcohol) and poly(acrylamide phenyl boronic acid-*co*-*N*–vinylcaprolactam) layers on porous poly(lactic-*co*-glycolic acid) particles loaded with insulin were used by Wu et al. [279] for controlling the blood sugar levels in diabetic mice. The treatment with capsules resulted in an effective control of the levels of sugar in blood for at least 18 days due to the sustained release of the insulin triggered for the glucose concentration. This is a significant improvement compared to the result found for the bare poly(lactic-*co*-glycolic acid) particles, which could provide regulation of the sugar levels for only seven days.

Balabushevich et al. [280] designed capsules loaded with insulin and coated by dextran sulphate and chitosan, which allowed a sustained release of the insulin at pH of around 7.4, providing a fast decrease in the glucose levels (1 h after the oral administration of the formulation). Furthermore, the addition of protease inhibitors to the capsules avoids the proteolysis of the encapsulated insulin, ensuring the delivery of active insulin in the intestine. Verma et al. [281] designed capsules loaded with insulin by combining layers of sodium alginate and vitamin B12 grafted chitosan, trying to take advantage of the role of the vitamin B12 in the control of the sugar levels in blood. The release of insulin triggered by the pH resulted in a significant increase in the insulin bioavailability and sustained hypoglycemic effects up to 12 h after the administration with respect to the multilayers without vitamin B12, evidencing the role of this component due to its pH sensitivity and targeting of the capsules. Furthermore, the mucoadhesive character of chitosan, and its ability to open tight junctions, enable the fabrication of pH-sensitive platforms for the oral delivery of insulin.

Song et al. [282] fabricated LbL PEMUCs for the alternate assembly of chitosan and heparin layers on particles of insulin and chitosan capped by the adsorption of poly(ethylenglycol) on the external surface avoiding the biofouling processes. Thus, it is possible to enhance the stability and availability of insulin in biological environments. Furthermore, the oral administration of the capsules provides a fast decrease in the sugar levels in blood, with good intestinal adsorption. Table 4 summarizes some examples of LbL capsules with potential use in diabetes treatment.

## 9. Layer-by-Layer Capsules for Theranostic Purposes

Theranosis is referred to the use of devices combining therapeutic and diagnostic utilities. This involves the use of nanotools to simultaneously obtain molecular and cellular information related to the disease, allowing for a personalized treatment. This requires combining both an imaging probe and a therapeutic agent in a single platform [283], which can be attained by taking advantage of the modularity of LbL for the assembly of hierarchical multifunctional structures using different molecular or colloidal entities [144].

Wang et al. [284] prepared theranostic LbL capsules by decorating calcium carbonate particles by the alternate deposition of poly-(l-ornithine) and fucoidan, which were loaded with doxorubicin, taking advantage of its attractive interactions with fucoidan. The obtained PEMUCs evidenced a sustained release of the encapsulated drug, inducing a very efficient inhibition of the proliferation of carcinogen cells (MCF-7 cells) without any cytotoxicity against healthy cells. LbL capsules including layers of PLL and folic acid, or a mixture of folic acid and folate antimetabolites, were tested as a theranostic device to examine their uptake and cytotoxicity in CT26 murine colorectal cancer cells [285]. According to fluorescence microscopy images, the cellular uptake of the capsules is enhanced when the capping layer contains folic acid or a mixture of folic acid and folate antimetabolites. Furthermore, the internalized capsules present a strong cytotoxicity against carcinogen cells with limited impact on healthy cells.

Svenskaya et al. [286] designed different types of polyelectrolyte capsules loaded with magnetite particles as NMR imaging contrast agents and for magnetically driven targeting, and found that the loading of PEMUCs with magnetite particles allows the manufacture of non-cytotoxic platforms for magnetic resonance imaging and specific targeting in tumor cells. These capsules contain magnetic nanoparticles within the inner cavity and embedded within the polyelectrolyte shell, and provide enhanced in vivo targeting in tumor cells under the action of external applied magnetic fields, offering a good contrast for tissue visualization. Furthermore, the inclusion of specific anticancer drugs contributes to the manufacturing of high power theranostic devices. Szczepanowicz et al. [287] proposed the preparation of nanocapsules for theranosis by coating oil droplets containing drugs with a hybrid shell containing poly-l-glutamic acid, poly-(l-lysine), and Fe_2_O_3_ nanoparticles. This type of capsule offers a good contrast for NMR imaging. Furthermore, capping the capsules with a pegylated polyelectrolyte ensures their stability in biological media, avoiding biofouling phenomena. The modification of the external surface of PEMUCs with specific polymers, such as chitosan, was proposed by Hanafy [288] for enhancing the theranostic power of LbL capsules. Thus, the combination of chitosan and folic acid in the capping layer enhances the capsule adhesion to the target cell, resulting in a better diagnosis of breast cancer and contributing to a more localized therapeutic effect of the encapsulated curcumin.

**Figure 12:**
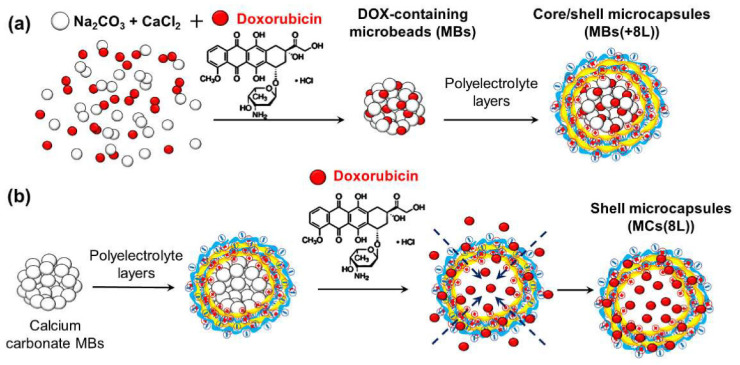
Sketch of the fabrication for LbL of different types of capsules for theranosis purposes. (**a**) Co-precipitation method. (**b**) Direct drug adsorption. MB: Microbeads, MC: Microcapsules. Reprinted from Kalinechenko et al. [36], with permission under Open access CC BY 4.0 license, https://creativecommons.org/licenses/by/4.0/ (accessed 20 January 2022).

Kalinechenko et al. [36] developed size-homogenous (PAH-PSS)_n_ PEMUCs with different structures (core/shell and shell types) loaded with doxorubicin as an anticancer drug, and fluorescent quantum dots following the procedures schematized in Figure 12.

The results by Kalinechenko et al. [36] evidenced that (PAH-PSS)_n_ capsules provide a sustained release of the encapsulated doxorubicin at pH 7.4, with the decrease in the pH resulting in a burst release, probably mediated by the erosion of the capsules. Furthermore, the different permeability of the capsules depending on their structure also influences the release profile. Thus, the release from microbeads (MBs) containing the drug embedded within a calcium carbonate core is slower than that from microcapsules (MCs) in which the drug is distributed within the inner cavity of the capsule and the shell. This leads to a situation in which drug release from MBs is more easily induced by erosion of the layers than as a result of the permeability changes. Furthermore, the inclusion of quantum dots allows the acquisition of bright fluorescence capsules that can be exploited for diagnosis using fluorescence imaging.

Ultrasound irradiation is also an alternative that can be exploited for theranosis, as shown by the results of Chen et al. [289]. They prepared multilayered capsules combining tannic acid and poly(*N*-vinylpyrrolidone) and loaded with doxorubicin, and showed that this type of capsule provides a high contrast for imaging using brightness and harmonic modes upon the irradiation with low-power ultrasound (∼100 mW/cm^2^), allowing their use for diagnostics. The diagnostic power can be tuned by modifying the number of layers, the properties of the multilayers, or the nature of the assembled polymers. In particular, the increase in the shell rigidity provides a significant enhancement of the imaging contrast, which is also increased as the drug concentration is increased. Furthermore, the release of doxorubicin from the capsules can be triggered upon irradiation with high-power ultrasound (>10 W/cm^2^), allowing the use of the capsules as a therapeutic tool, as evidenced by their ability to induce a 97% cytotoxicity on MCF-7 human cancer cells with only 50% of released doxorubicin. The combination of tannic acid and poly(*N*-vinylpyrrolidone) was also exploited for dual triggering of theranostic biocompatible platforms, including iron nanoparticles as a contrast agent for magnetic resonance imaging, and encapsulated doxorubicin, which can be released upon irradiation with ultrasound [290]. This type of capsule presents an improved circulation in the blood stream in relation to free doxorubicin, providing a 16-fold increase for the doxorubicin targeting in breast cancer cells. Dual triggering was also used by Kozlovskaya et al. [291] with multilayers of tannic acid and poly(*N*-vinylpyrrolidone) containing doxorubicin and chelated on their surface with ^89^Zr. This type of system offers very good properties for in vivo imaging via positron emission tomography (PET) and doxorubicin targeting tumors cell through therapeutic ultrasound-triggered release. Furthermore, this type of capsule offers good stability and long-term duration of its imaging and therapeutic properties. This has pushed further developments of capsules combining PET imaging and therapeutic approaches. Muslimov et al. [292] prepared multilayered capsules combining tannic acid and bovine serum albumin and chelated with ^89^Zr for PET imaging, which were radiolabeled using ^225^Ac for antitumoral treatment. This type of capsule demonstrated radiochemical stability and good ability for the retention of the ^225^Ac and its daughter isotopes (^221^Fr and ^213^Bi) [293]. The in vivo injection of the capsules results in a significant accumulation of the capsule in the tumoral region, as evidenced by PET analysis, which agrees with the radiometry values. Furthermore, the capsules containing the antitumoral preparation do not affect healthy tissues after treatment, whereas they lead to a significant inhibition of tumor growth, resulting in a prolonged survival of individuals upon treatment.

Novoselova et al. [294] designed more sophisticated theranostic platforms than those discussed above. They combined magnetic nanoparticles and doxorubicin in LbL capsules, and a fluorescent dye. This enables an enhanced contrast for magnetic resonance experiments while facilitating the magnetic targeting of the capsules. Furthermore, the magnetic particles allow a remote-controlled release of the encapsulated doxorubicin upon the application of high-intensity focused ultrasound. The presence of the magnetic particles combined with the dyes also enables evaluation of the release of the drug by the optoacoustic signal and fluorescent tomography. Therefore, this type of carrier is a very useful alternative for simultaneous imaging and focal therapy. This is supported by the higher toxicity induced for the encapsulated doxorubicin in relation to its free form. Table 5 summarizes some examples of LbL systems designed for theranostic purposes.

The above discussion evidences the power of LbL capsules as theranostic agents in externally triggered chemotherapy, presenting a high encapsulation efficiency and biocompatibility, and tunable drug release.

## 10. Layer-by-Layer Capsules for Hyperthermia Treatments

Hyperthermia relies on the treatment of tumors based on the localized heating of cells by the application of an external electromagnetic field to magnetic probes incorporated in the diseased tissues or organs. This type of treatment ensures a deep tissue penetration, and a high selectivity for killing cells without harmful effects on the surrounding tissues [295,296].

Zharkov et al. [297] proposed the design of hyperthermia platforms based on LbL capsules formed with dextran sulphate and poly-(l-arginine), and embedded iron oxide particles within the LbL shell. The obtained capsules were taken up by human fibroblasts upon the application of an alternating magnetic field, inducing a stronger cytotoxic effect than that of free magnetic nanoparticles. Furthermore, the use of capsules ensures a local hyperthermia with minimal heating of the environment. Zuyzin et al. [298] designed hyperthermia platforms for a sustained and predictable heating dose inside biological matrices. For this purpose, they assembled PEMUCs loaded with iron oxide cubes in the inner cavity of capsules formed by the alternate assembly of PAH and PSS. This allows retaining a certain degree of the particles’ mobility, minimizing their aggregation, which enables the preservation of the heating power of magnetic particles upon their magnetization. After capsule uptake, the specific adsorption rate of particles is enhanced in relation to that of free particles, indicating a very promising platform for hyperthermia applications.

Cristofolini et al. [299] designed highly efficient hyperthermia platforms by embedding Fe_3_O_4_ particles in (PLL-PGA)_n_ capsules, which were decorated with poly(ethylenglycol) chains grafted to the external surface to ensure the cellular uptake. The obtained capsules can be guided and concentrated at the target tissue by applying a radio frequency magnetic field, which can also be exploited for magnetic hyperthermia treatments as a result of the magnetization-induced heating.

## 11. Conclusions

This review highlighted the potential of PEMUCs as drug delivery and diagnosis platforms, providing an updated perspective on the most novel advances that enable application of LbL microcapsules in the field of biomedicine. These advances are being pursued due to the versatility and simplicity of the LbL method for the controlled fabrication of supramolecular systems with a broad range of molecules, including polymers, colloids, biomolecules, and even cells. This approach can thus be exploited for the fabrication of high-performance nanosystems combining different drugs, and due to the multiple possibilities offered by the LbL method for the fabrication of PEMUCs with different structures and a broad range of compositions. This allows the manufacture of systems with patient-specific agents for precision medicine, providing new opportunities for the development of new diagnoses and therapeutic tools. However, further research on the potential applications of PEMUCS to this field is currently in progress. In particular, research is currently examining the optimization of the biocompatibility of manufactured PEMUCs because most of the proposed options involve the use of components that are toxic themselves.

## Figures and Tables

**Table 1:** Summary of multilayered capsules used for antibiotic release.

Multilayer Components	Encapsulated Drug	Loading	Release	Reference
PAH and PSS	gentamicin	Pre-loading (trapping in silicon dioxide templates)	pH-induced	Al Thaher [239]
PLL and HA	vancomycin *Polyhexamethylene biguanide*	Post-loading (direct incubation)	pH-induced (bacteria protease-triggered)	Craig et al. [240]
branched polyethyleneimine and tannic acid	tobramycin gentamicin polymyxin B	Pre-loading (combined with tannic acid layers)	pH-induced	Zhuk et al. [241]
diethylaminoethyl-dextran hydrochloride and dextran sulphate	kamayicin	Post-loading (direct incubation)	pH-induced	Pawlak et al. [242]

**Table 2:** Summary of multilayered capsules used for antitumoral drug release.

Multilayer Components	Encapsulated Drug	Loading	Release	Type of Tumor	Reference
HA modified with β-cyclodextrin and PLL	paclitaxel	Pre-loading (host-guest interactions with β-cyclodextrin)	environmentally triggered (hyaluronidase enzyme action)	breast cancer (MDA-MB-231 cells)	Jing et al. [244]
alginic acid and protamine	*cisplatin*	Post-loading (direct incubation)	pH-induced	human breast cancer (MCF-7 cells) human cervical cancer (HeLa cells) human ovarian cancer (SKOV-3 cells) human epithelial colorectal adenocarcinoma (CACO-2 cells)	Vergaro et al. [245]
chitosan and alginate	doxorubicin	Post-loading (direct incubation with pH changes)	pH-induced (acidic conditions)	human breast cancer (MCF-7 and MCF-7/ADR cells)	Shen et al. [246]
poly(l-arginine) and dextran sulphate	doxorubicin	Post-loading (direct incubation with temperature changes)	pH-induced	human breast cancer (MCF-7 and MCF-7/ADR cells)	Trushina et al. [247]
PAH and PSS	doxorubicin	Post-loading (direct incubation with pH changes)	pH-induced (acidic conditions)	without tests	Yang et al. [249]
PLL and PGA	*cisplatin*	Pre-loading (combined with PLL layers)	pH-induced (acidic conditions) redox induced (reductive conditions)	colon cancer (CT-26 cells)	Zhou et al. [250]
protamine and heparin	doxorubicin	Post-loading (direct incubation with pH changes)	pH-induced (erosion in acid medium)	human breast cancer (MCF-7 cells)	Radhakrishnan et al. [251]
poly(*N*-vinylpyrrolidone) and poly(methacrylic acid)	iminoquinone	Pre-loading (included within the template)	redox induced (reductive conditions induced by glutathione)	hepatocellular carcinoma (HepG2 and Huh7cells)	Xue et al. [252]
PDADMAC and PSS	doxorubicin	Post-loading (direct incubation)	pH-induced	human cervical cancer (HeLa cells)	Kittitheeranun et al. [253]

**Table 3:** Summary of multilayered capsules used for gene therapy.

Multilayer Components	Genetic Material	Loading	Release	Reference
PAH and PSS dextran sulphate and poly-(l-arginine)	DNA plasmid	Pre-loading (trapping in CaCO_3_ templates)	environmentally triggered (trip sine action)	Santos et al. [258]
PAH, protamine sulfate and dextrane sulfate	DNA plasmid	Pre-loaded (incorporated as a layer)	environmentally triggered (erosion process)	Reibetanz et al. [270]
poly(l-arginine) and dextran sulphate	mRNA siRNA	Pre-loading (trapping in CaCO_3_ templates)	environmentally triggered	Tarakanchikova et al. [271]
poly(l-arginine) and dextran sulphate	mRNA	Pre-loading (trapping in CaCO_3_ templates)	environmentally triggered	Kakran et al. [272]
poly(ethyleneimine) or poly(amino pentanol) and DNA	DNA	Pre-loaded (incorporated as a layer)	environmentally triggered	Xie et al. [273]
poly(ethyleneimine) and HA	siRNA	Pre-loaded (incorporated as a layer)	environmentally triggered	Koenig et al. [274]

**Table 4:** Summary of multilayered capsules used for diabetes treatment.

Multilayer Components	Loading	Release	Reference
chitosan and insulin	Pre-loading (insulin incorporated as a layer)	pH-induced (acidic condition)	Zhang et al. [278]
poly(vinyl alcohol) and poly(acrylamide phenyl boronic acid-*co*-*N*-vinylcaprolactam)	Pre-loading (trapping in poly(lactic-*co*-glycolic acid) templates)	environmentally triggered (glucose levels)	Wu et al. [279]
chitosan and dextran sulphate	Pre-loading (incorporated as core)	pH-induced	Balabushevich et al. [280]
vitamin B12 grafted chitosan and sodium alginate	Pre-loading (incorporated in the template)	pH-induced	Verma et al. [281]
chitosan and heparin	Pre-loading (as hybrid core with chitosan)	pH-induced	Song et al. [282]

**Table 5:** Summary of multilayered capsules used for theranostic purposes.

Multilayer Components	Drug	Diagnostic Probe	Purpose	Reference
poly-(l-ornithine) and fucoidan	doxorubicin (pre-loaded combined with fucoidan layers)	fluorescent probe	anticancer	Wang et al. [284]
PLL and folic acid (or mixture of folic acid and folate antimetabolites)	folic acid (or mixture of folic acid and folate antimetabolites) included as layers	fluorescent probe	anticancer	Rosch et al. [285].
poly-l-arginine and dextran sulfate	magnetite particles (pre-loaded within the template or adsorbed as layers)	magnetite particles (NMR imaging)	anticancer	Svenskaya et al. [286]
PLL and poly-l-glutamic acid	doxorubicin (preloaded in the oil core of the emulsion droplets used as template)	Fe_2_O_3_ nanoparticles (NMR imaging)	anticancer	Szczepanowicz et al. [287]
dextran, protamine, chitosan, and folic acid	curcumin (post-loaded by direct incubation)	fluorescent probe (dextran marked with rhodamine as layer)	anticancer	Hanafy [288]
PAH and PSS	doxorubicin (pre-loaded in CaCO_3_ templates)	quantum dots (fluorescence imaging)	anticancer	Kalinechenko et al. [36]
tannic acid and poly(*N*-vinylpyrrolidone)	doxorubicin (pre-loaded in the template)	capsule itself ultrasound imaging	anticancer	Chen et al. [289]
tannic acid and poly(*N*-vinylpyrrolidone)	doxorubicin (pre-loaded in silicon dioxide templates)	iron nanoparticles (NMR imaging)	anticancer	Alford et al. [290]
tannic acid and poly(*N*-vinylpyrrolidone)	doxorubicin (post-loaded by direct incubation)	^89^Zr (PET imaging)	anticancer	Kozlovskaya et al. [291]
tannic acid and bovine serum albumin	^225^Ac (pre-loading by radiolabeling)	^89^Zr (PET imaging)	anticancer	Muslimov et al. [292]
poly-l-arginine and dextran sulfate	doxorubicin (pre-loaded in CaCO_3_ templates)	fluorescent probes and magnetic nanoparticles (fluorescent tomography and optoacoustic signal)	anticancer	Novoselova et al. [294]

## Data Availability

This work does not contain any associated data.
